# The histone deacetylase Hda1 affects oxidative and osmotic stress response as well as mycoparasitic activity and secondary metabolite biosynthesis in *Trichoderma atroviride*

**DOI:** 10.1128/spectrum.03097-23

**Published:** 2024-02-09

**Authors:** Verena Speckbacher, Daniel Flatschacher, Nora Martini-Lösch, Laura Ulbrich, Clara Baldin, Ingo Bauer, Veronika Ruzsanyi, Susanne Zeilinger

**Affiliations:** 1 Department of Microbiology, Universität Innsbruck, Innsbruck, Austria; 2 Umweltmonitoring und Forensische Chemie, Hochschule Hamm-Lippstadt, Hamm, Germany; 3 Institute of Molecular Biology, Biocenter, Medical University of Innsbruck, Innsbruck, Austria; 4 Breath Research Institute, Universität Innsbruck, Dornbirn, Austria; Universidade de Sao Paulo, Sao Paolo, Brazil

**Keywords:** histone deacetylase, *Trichoderma atroviride*, secondary metabolites, mycoparasitism, oxidative and osmotic stress, volatile organic compounds, inhibition, RNA sequencing, transcriptome, HPTLC

## Abstract

The mycoparasitic fungus *Trichoderma atroviride* is applied in agriculture as a biostimulant and biologic control agent against fungal pathogens that infest crop plants. Secondary metabolites are among the main agents determining the strength and progress of the mycoparasitic attack. However, expression of most secondary metabolism-associated genes requires specific cues, as they are silent under routine laboratory conditions due to their maintenance in an inactive heterochromatin state. Therefore, histone modifications are crucial for the regulation of secondary metabolism. Here, we functionally investigated the role of the class II histone deacetylase encoding gene *hda1* of *T. atroviride* by targeted gene deletion, phenotypic characterization, and multi-omics approaches. Deletion of *hda1* did not result in obvious phenotypic alterations but led to an enhanced inhibitory activity of secreted metabolites and reduced mycoparasitic abilities of *T. atroviride* against the plant-pathogenic fungi *Botrytis cinerea* and *Rhizoctonia solani*. The *∆hda1* mutants emitted altered amounts of four volatile organic compounds along their development, produced different metabolite profiles upon growth in liquid culture, and showed a higher susceptibility to oxidative and osmotic stress. Moreover, *hda1* deletion affected the expression of several notable gene categories such as polyketide synthases, transcription factors, and genes involved in the HOG MAPK pathway.

**IMPORTANCE:**

Histone deacetylases play crucial roles in regulating chromatin structure and gene transcription. To date, classical—Zn^2+^ dependent—fungal histone deacetylases are divided into two classes, of which each comprises orthologues of the two sub-groups Rpd3 and Hos2 and Hda1 and Hos3 of yeast, respectively. However, the role of these chromatin remodelers in mycoparasitic fungi is poorly understood. In this study, we provide evidence that Hda1, the class II histone deacetylases of the mycoparasitic fungus *Trichoderma atroviride*, regulates its mycoparasitic activity, secondary metabolite biosynthesis, and osmotic and oxidative stress tolerance. The function of Hda1 in regulating bioactive metabolite production and mycoparasitism reveals the importance of chromatin-dependent regulation in the ability of *T. atroviride* to successfully control fungal plant pathogens.

## INTRODUCTION

*Trichoderma atroviride* is a worldwide-occurring, soil-inhabiting, filamentous fungus belonging to the division of Ascomycota. It has a necrotrophic mycoparasitic lifestyle and is antagonizing and preying on a broad range of fungal hosts. *T. atroviride* is a valuable biocontrol agent against plant pathogenic fungi and is acting beneficially on crop plants by enhancing plant growth in root and shoot as well as the plants resilience to biotic and abiotic stress factors (1). The mycoparasitic lifestyle implies that *Trichoderma* fungi are exposed to harsh environments where they, on the one hand, have to effectively sense the presence of potential host fungi and, on the other hand, have to compete and overcome the host’s defense mechanisms to finally succeed in the mycoparasitic attack. It is tempting to speculate that antifungal secondary metabolites substantially contribute to the success of the mycoparasitic fungus-fungus interaction, a hypothesis supported by an enrichment of secondary metabolism-associated genes in strong mycoparasites such as *T. atroviride* and *Trichoderma virens* compared to the only weakly mycoparasitic *Trichoderma reesei* and other saprophytic relatives (2, 3).

Many of the genes involved in secondary metabolite biosynthesis are part of large biosynthetic gene clusters comprising core enzymes such as non-ribosomal peptide synthetases (NRPSs), polyketide synthases (PKSs), or terpene synthases/cyclases, accessory enzymes (like cytochrome P450s, oxidoreductases, methyl transferases, etc.), and genes for transporters and transcription factors (4). Genome based *in silico* prediction of secondary metabolism-associated gene clusters and comparison with the metabolites produced under routine laboratory conditions showed that most gene clusters are not expressed and remain silenced under these conditions (3, 4). This may be related to the fact that secondary metabolite biosynthetic gene clusters (BGCs) often are localized in sub-telomeric regions of the chromosomes (5), which are characterized by a high degree of the condensed and transcriptionally inactive heterochromatin. Hence, the expression of these genes is tightly regulated in fungi, and several of them are epigenetically controlled with having repressive chromatin marks during primary metabolism-favoring conditions (6).

DNA accessibility is affected by posttranslational modifications which define the state of chromatin that can be either loosely packed and hence transcriptionally active (euchromatin) or more densely packed and transcriptionally silent (heterochromatin). Acetylation of histones is among the most abundant modifications. This is achieved by histone acetyltransferases (HATs), which add acetyl groups, and histone deacetylases (HDACs), which are responsible for their removal. In general, histone acetylation is associated with active gene transcription, while histone deacetylation results in gene silencing (7, 8). In fungi, histone acetylation has been shown to be crucial for transcriptional regulation of various processes, including secondary metabolism and virulence. (9, 10). Consequently, the overexpression of HATs and the deletion of HDACs, respectively, is a promising approach to activate the expression of secondary metabolism-associated silent genes and gene clusters (6–8).

“Classical”—Zn^2+^ ion binding—fungal lysine deacetylases can be assigned to class I (Rpd3 family comprising the enzymes Rpd3 and Hos2) and class II (Hda1 family comprising the enzymes Hda1 and Hos3) HDACs, based on their sequence similarity to the respective *Saccharomyces cerevisiae* orthologues (11–15). Functional characterization of the class I HDAC Hda-2 in *T. atroviride*—the orthologue of *S. cerevisiae* Hos2—was described to regulate growth, conidiation, blue light perception, and oxidative stress response (16). In *Aspergillus nidulans*, the class II HDAC encoding gene *hdaA* is accountable for the majority of HDAC activity (17), and its deletion led to the transcriptional activation of gene clusters for sterigmatocystin and penicillin production and a de-repression of telomere-proximal secondary metabolite gene clusters (18). In *Aspergillus fumigatus*, *∆hdaA* mutants showed not only an up-regulation of the production of several secondary metabolites but also a down-regulation of gliotoxin biosynthesis (19). Furthermore, Hda1 was described to regulate secondary metabolite production in *Magnaporthe oryzae* and *Fusarium asiaticum* (20) and to play essential roles in the virulence of the plant pathogen *Fusarium fujikuroi* (21).

Consequently, we hypothesized that the class II HDAC Hda1 of *T. atroviride* plays a major role in (i) the regulation of secondary metabolism-associated genes and gene clusters and, hence, the biosynthesis of secondary metabolites as well as (ii) the mycoparasitic activity of *T. atroviride*. We functionally characterized *hda1* by generating and phenotypically analyzing respective *T. atroviride* deletion mutants, including a multi-omics approach. The latter comprised gas chromatography-ion mobility spectrometry (GC-IMS) analysis of volatile organic compounds (VOC), high-performance thin-layer chromatography (HPTLC) analysis of secreted water-soluble metabolites and comparative RNA sequencing to monitor global Hda1-dependent alterations of the fungal transcriptome. We provide evidence that *hda1* deletion significantly increases the antifungal effect of the secreted metabolite cocktail but, at the same time, impairs the mycoparasitic capabilities of *T. atroviride* during the direct interaction with host fungi. Furthermore, Hda1 affected the response of the fungus to oxidative and osmotic stress and seemed to act as a major regulator on a transcriptional and metabolic level in *T. atroviride*.

## RESULTS

### Identification of Hda1 in *T. atroviride* and generation of deletion mutants

BLASTp analysis of functionally characterized Hda1 proteins of various fungal species (Table S1) identified the respective *T. atroviride* P1 orthologue as Triatrov1_386002. *T. atroviride* Hda1 shows a protein sequence identity of 48% to yeast Hda1p. In a phylogenetic tree (Fig. S1), the HDAC sequences of *Alternaria alternata*, *A. fumigatus*, *B. cinerea*, *Candida albicans*, *Cladosporium fulvum*, *F. fujikuroi*, *Neurospora crassa*, *S. cerevisiae*, and *Ustilago maydis* clustered into the four HDAC subfamilies Hda1, Hos2, Hos3, and Rpd3, with Hda1 of *T. atroviride* being clearly affiliated to the Hda1 subfamily.

For the functional characterization of Hda1 in *T. atroviride*, deletion mutants were generated by targeting the *hda1* gene locus via homologous recombination with a hygromycin B resistance conveying split marker deletion cassette. Two rounds of transformation resulted in 96 and 29 independent transformants, respectively, which were subsequently purified to mitotic stability by three rounds of single spore isolation. All transformants were screened via a PCR-based strategy targeting both flanking regions (Fig. S2A) that lead to the identification of three independent deletion mutants named *∆hda1A*, *∆hda1B*, and *∆hda1C* (Fig. S2B).

### *Hda1* gene deletion affects the deacetylation of histones H3 and H4

**Fig 1:**
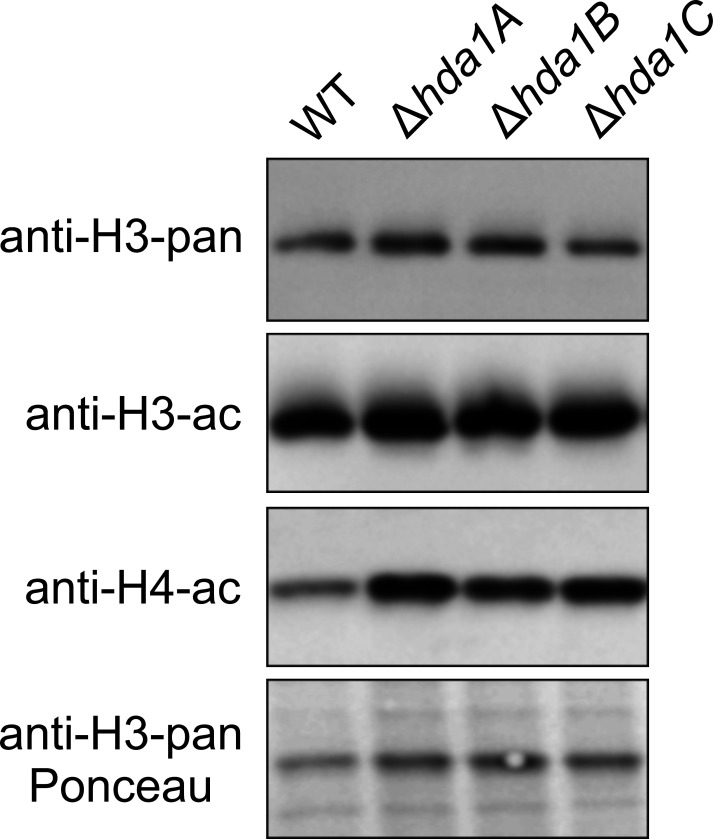
Acetylation levels of histones H3 and H4 in the *T. atroviride* ∆*hda1* mutants and the WT. Extracted proteins were separated on 16% polyacrylamide gels, and after blotting, the membranes were probed with anti-acetyl-histone H3 (anti-H3-ac) and anti-acetyl-H4 (anti-h4-ac) antibodies to detect the acetylation status. Ponceau staining and probing with an anti-histone H3 pan (anti-h3-pan) antibody were used to prove consistent loading and as reference for relative quantification, respectively.

Since loss of Hda1p activity in yeast has been shown to result in histone hyperacetylation (22), we analyzed the *in vivo* acetylation levels of the histones H3 and H4 in the *T. atroviride ∆hda1* mutants and the wild-type (WT) by Western blotting using acetyl-histone antibodies. In protein extracts obtained from the mutants, acetylation of both histones was increased compared to extracts derived from the WT suggesting that Hda1 is involved in the deacetylation of histones H3 and H4 in *T. atroviride* (Fig. 1).

### *Hda1* gene deletion decelerates radial growth of spore-inoculated cultures

**Fig 2:**
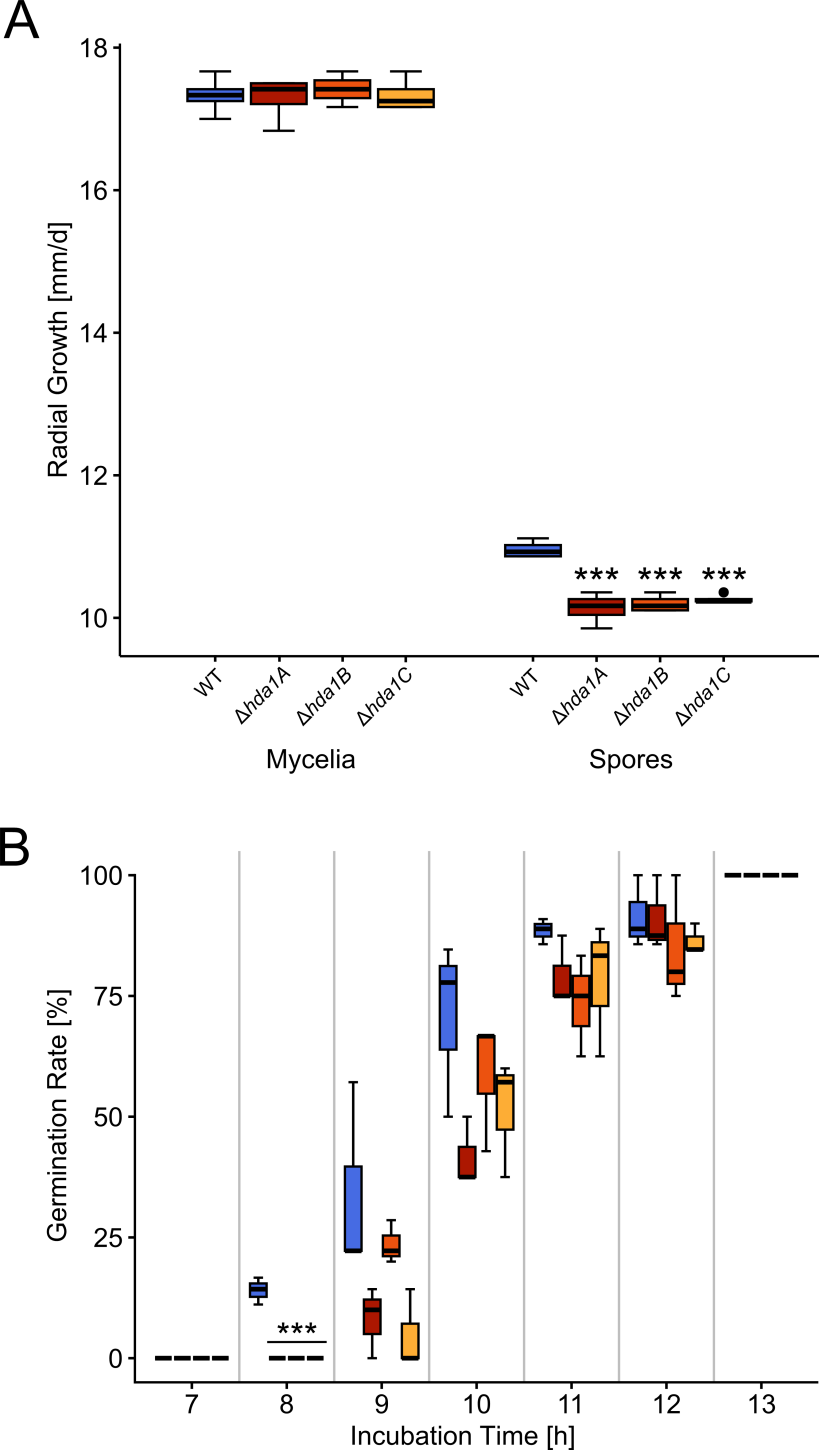
Radial growth rates of colonies developing from inoculation with mycelia or spores and germination rate of spores of the wild-type (WT) and ∆*hda1* mutants. (**A**) Whisker-box plot comparing the radial growth rates (mm/d) of solid cultures of the WT and the *∆hda1* mutants grown from mycelial plugs (Mycelia) or from fresh spore suspensions (1 * 10^5^ spores; Spores). The radial growth rate was determined after 48 h of growth under 12-/12-h light-dark conditions at 25°C. Asterisks indicate statistically significant differences between each *hda1* mutant and the WT within each inoculation type (*n* = 4, *P < 0.05, **P < 0.01, ***P < 0.001, ****P < 0.0001, details of the statistical evaluation are given in Table S2). (**B**) Germination rates of the WT (blue boxes) and the deletion mutants *∆hda1A*, *∆hda1B*, and *∆hda1C* (red, orange to yellow boxes; left to right) grown in liquid media from fresh spore suspension (5 * 10^6^ spores/mL). The germination rate was determined every hour between 7 and 13 h of growth at 25°C and 250 rpm. Asterisks indicate statistically significant differences between ∆*hda1* mutants and the WT within each measurement timepoint (*n* = 3, *P < 0.05, **P < 0.01, ***P < 0.001, details of the statistical evaluation are given in Table S2).

The radial growth rate of fungal colonies of both, the WT and the mutants, strongly differed between growth after mycelia-covered plug inoculation and growth after inoculation with spore suspensions (Fig. 2A). With mean values of 17.33, 17.29, 17.42, and 17.33 mm/d, respectively, the radial growth rates of solid cultures grown from mycelial inoculation were highly similar between the WT and the three deletion mutants ∆*hda1A*, ∆*hda1B*, and ∆*hda1C*. In contrast, the radial growth rates of solid cultures grown from spores were significantly reduced in the mutants compared to the WT, with 10.14, 10.20, and 10.26 compared to 10.96 mm/d, respectively. The germination rate of ∆*hda1* spores was significantly delayed 8 h after inoculation and afterward reached levels similar to the WT (Fig. 2B).

### *Hda1* gene deletion impairs the mycoparasitic interaction but increases the inhibitory effect of secreted antifungal metabolites

**Fig 3:**
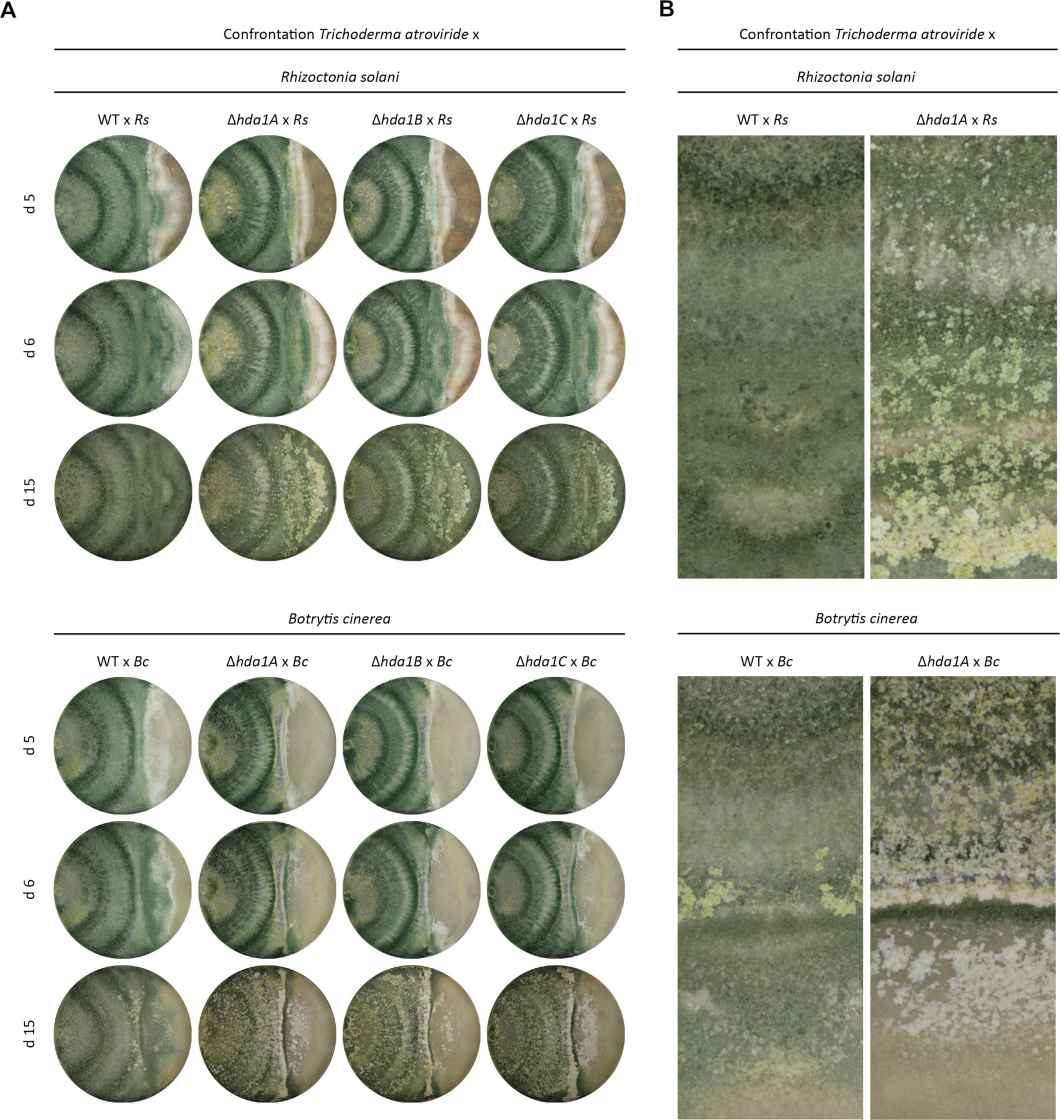
Confrontation assay for the assessment of the mycoparasitic activity. (**A**) Mycoparasitic interaction of the *T. atroviride* wild-type (WT) and the three mutants *∆hda1A*, *∆hda1B*, and *∆hda1C* confronted (x) with *Rhizoctonia solani* (Rs) or *Botrytis cinerea* (Bc). The plates were inoculated with mycelial plugs and incubated at 25°C under 12-/12-h light-dark conditions for a total time span of 15 days (15 d). Photos were taken after 5, 6, and 15 days (d 5, 6, 15) of growth. One representative photo out of four biological replicates (*n* = 4) is shown. (**B**) Enlarged image details of the hyphal structures in the mycoparasitic interaction of the *T. atroviride* WT and the *∆hda1A* mutant (upper area) confronted (**X**) with the host fungi *Rhizoctonia solani* (Rs) or *Botrytis cinerea* (Bc) (lower area) after 15 days of inoculation. One representative photo out of four biological replicates (*n* = 4) is shown.

In dual confrontation assays with the plant pathogens *R. solani* or *B. cinerea*, an impaired overgrowth activity of *∆hda1A*, *∆hda1B*, and *∆hda1C* compared to the WT became obvious after five days of cultivation (Fig. 3A). Furthermore, characteristic alterations in the macroscopic colony morphology developed in the deletion mutants at late interaction stages. In confrontation with *R. solani*, *hda1* deletion led to a delayed but complete overgrowth of the host fungus, whereas in the direct interaction with *B. cinerea*, the mutants were not able to completely overgrow the fungal host within 15 days. ∆*hda1* mutants developed whitish, hyphal crusts which started to emerge from 10 days on. In the interaction with *R. solani*, those structures arose mostly in the direct interaction zone and the mycoparasitic overgrowth zone, whereas in confrontation with *B. cinerea*, the structures were mainly formed in the direct interaction zone and the mycelia of the *hda1* deletion mutants themselves (Fig. 3B).

**Fig 4:**
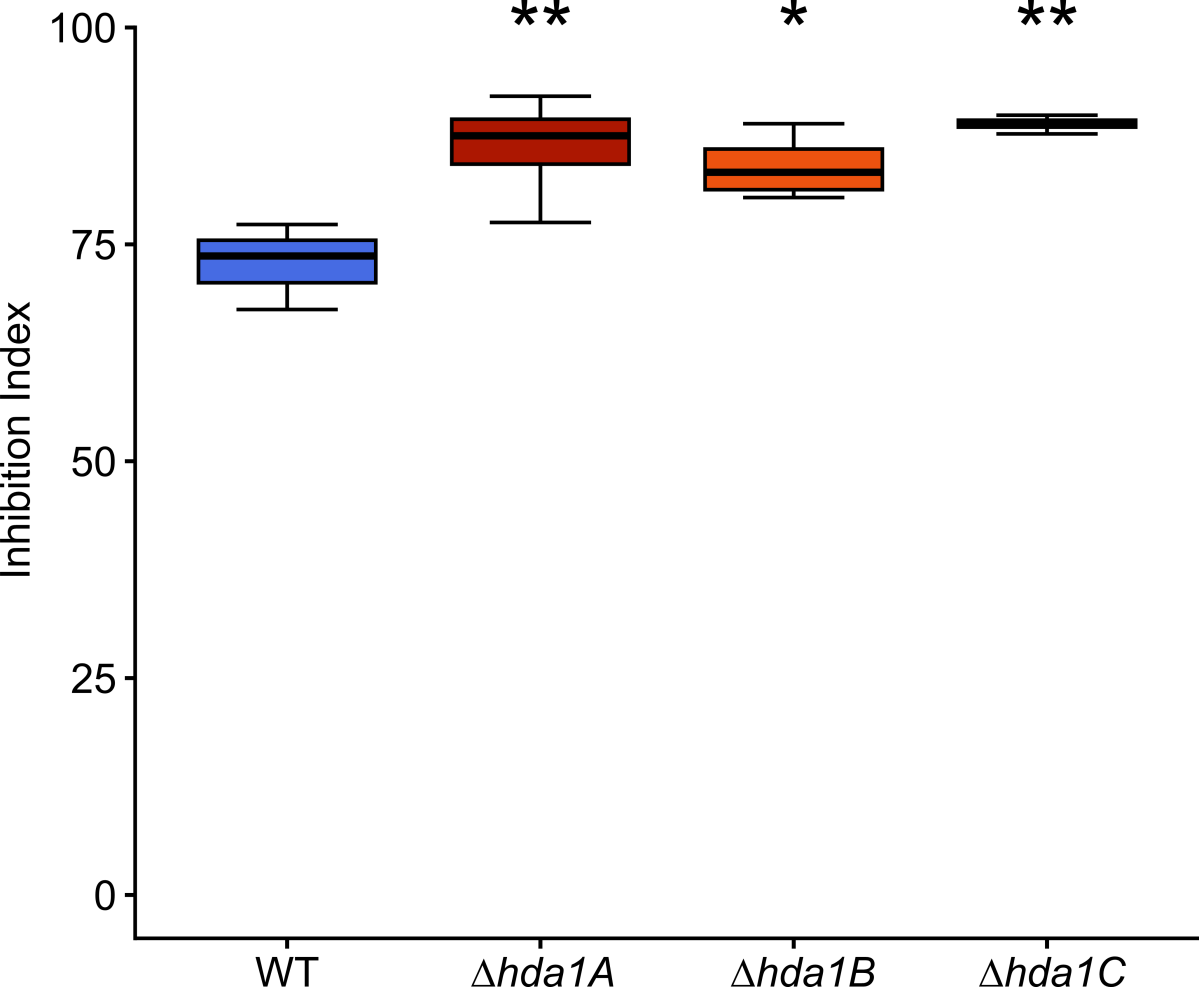
Inhibitory activity of diffusible metabolites secreted by the *T. atroviride* wild-type (WT) and ∆*hda1* mutants on *Botrytis cinerea*. Index of inhibition mediated by diffusible metabolites secreted by the *T. atroviride* WT and its deletion mutants *∆hda1A*, *∆hda1B*, and *∆hda1C* on *B. cinerea* spore germination and growth. *T. atroviride* strains were grown on cellophane-covered PDA plates for 32.5 h, and the mycelia-covered membranes were removed. Afterward, *B. cinerea* spores (1 * 10^5^ spores/plate) were inoculated, and the plates were incubated for additional 44.5 h. Asterisks indicate statistically significant differences between each ∆*hda1* mutant and the WT (*n* = 4, *P < 0.05, **P < 0.01, ***P < 0.001). Details of the statistical evaluation are given in Table S2).

In contrast to the decreased mycoparasitic overgrowth abilities of the ∆*hda1* mutants, the inhibitory activity of diffusible metabolites secreted by *T. atroviride* on the germination and growth of *B. cinerea* was significantly enhanced (average increase of 13.5%) in the ∆*hda1* mutants compared to the WT (Fig. 4).

### *Hda1* gene deletion results in altered VOC emission

Since all three deletion mutants showed reproducible and comparable phenotypes and characteristics in the growth and confrontation assays, *∆hda1A* was randomly selected for detailed studies at the metabolic and transcriptomic levels.

**Fig 5:**
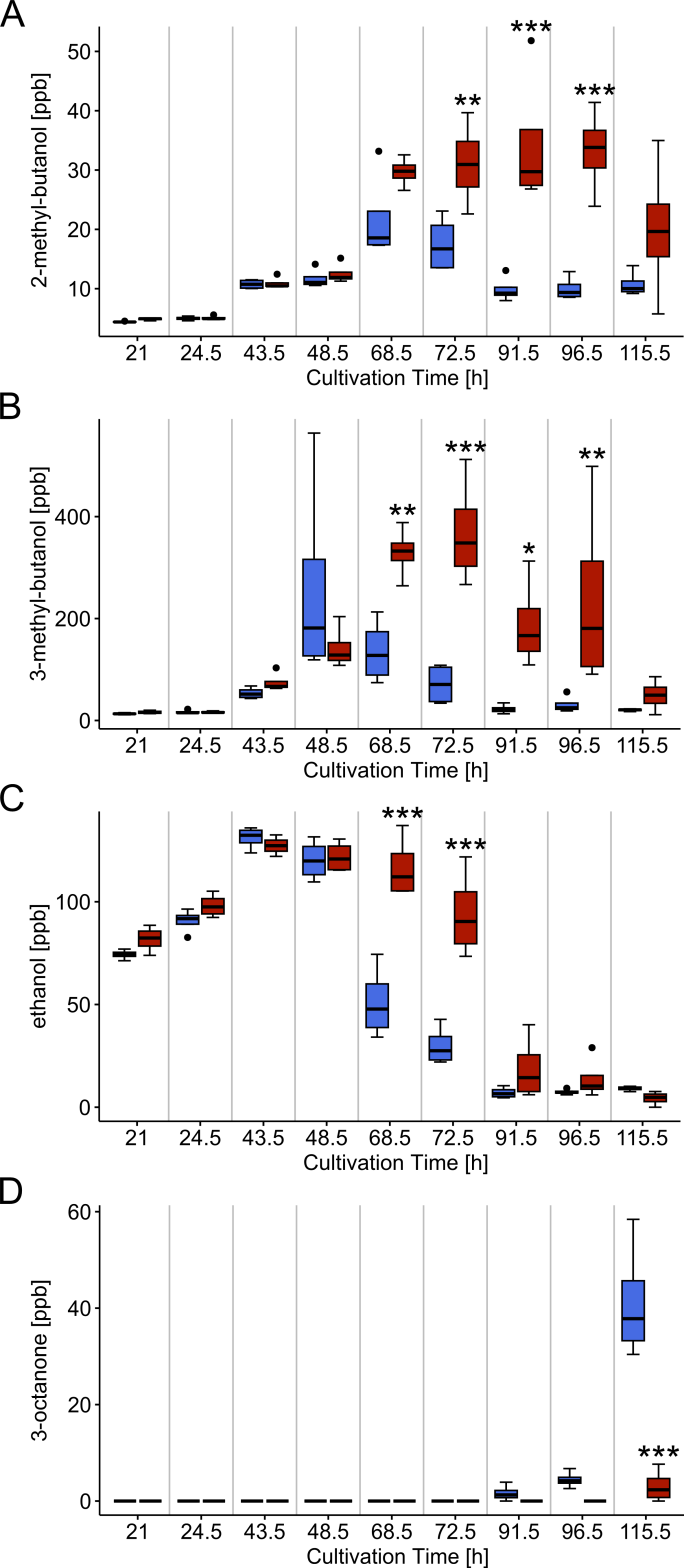
Volatile organic compound (VOC) emission by the *T. atroviride* wild-type (WT) and the ∆*hda1A* mutant along a cultivation period of 115.5 h. VOC concentrations (ppb) of 2-methyl-butanol (**A**), 3-methyl butanol (**B**), ethanol (**C**), and 3-octanone (**D**) emitted to the culture headspace by the *T. atroviride* WT (blue boxes) and the *∆hda1A* deletion mutant (red boxes). VOCs were measured at 21, 24.5, 43.5, 48.5, 68.5, 72.5, 91.5, 96.5, and 115.5 h of incubation via GC-IMS. Asterisks indicate statistically significant differences within each of the four VOCs between the *hda1A* mutant and the WT within each single measurement time point (*n* = 4, *P < 0.05, **P < 0.01, ***P < 0.001, ****P < 0.0001). Details of the statistical evaluation are given in Table S2).

Among the VOCs detected in GC-IMS, 2-methy-butanol, 3-methyl-butanol, 3-octanone, and ethanol were emitted to the culture headspace with characteristic and statistically significant concentration differences between the *∆hda1A* mutant and the WT. In the WT, 2-methyl-butanol emission started at 21 h, peaked after 68.5 h with a maximum mean value of 22 ppb, and then decreased again. The ∆*hda1A* mutant produced increasing amounts from 21 h on with 2-methyl-butanol levels peaking at a later time point with higher concentrations (34 ppb at 91.5 h; Fig. 5A). A similar emission curve and differences between the mutant and the WT were detected for 3-methyl-butanol. In the WT, the release of 3-methyl-butanol peaked after 48.5 h of growth with a maximum mean value of 261 ppb. In ∆*hda1A*, 3-methyl-butanol emission peaked after 72.5 h of cultivation with a maximum mean value of 369 ppb and thereafter decreased again with a second smaller peak of 238 ppb at 96.5 h of growth (Fig. 5B). Ethanol was emitted to the headspace already before the first measurement time point, where a concentration of around 80 ppb was detected in both, the WT and the deletion mutant. Ethanol levels increased until 43.5 h of cultivation up to approximately 130 ppb. Thereafter, the emission decreased until the end of cultivation. This decline was lower in the deletion mutant, which still emitted 94 ppb compared to 30 ppb released by the WT after 72.5 h of cultivation (Fig. 5C). In contrast to the other three VOCs, 3-octanone was exclusively detectable in the late cultivation phase and its levels were reduced in ∆*hda1A* compared to the WT. In the WT, 3-octanone emission started to increase after 91.5 h of cultivation and culminated in a maximum mean value of 41 ppb, whereas the deletion mutant released only 3 ppb at the latest measurement time point (Fig. 5D).

### *Hda1* gene deletion results in increased sensitivity to osmotic and oxidative stress

**Fig 6:**
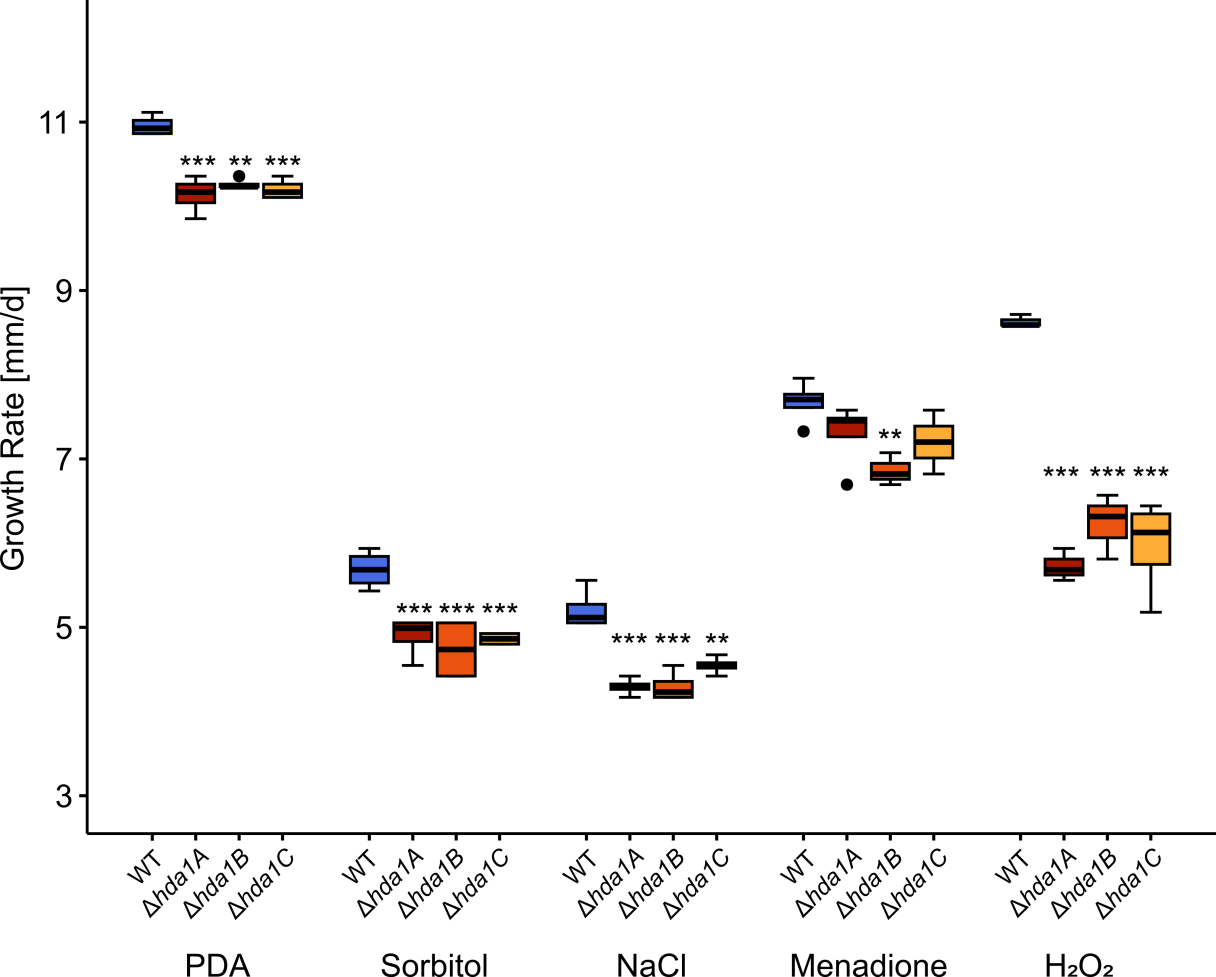
Influence of osmotic and oxidative stress on the radial growth rate of *T. atroviride* wild-type (WT) and ∆*hda1* mutants. The WT and the *∆hda1* mutants were cultivated on PDA, on PDA supplemented with sorbitol (700 mM), sodium chloride (NaCl; 500 mM) (both triggering osmotic stress), or menadione (50 mM) and hydrogen peroxide (H_2_O_2_; 2.5 mM) (the latter two triggering oxidative stress). Plates were point inoculated with 1 * 10^5^ spores, and fungal growth was evaluated after 47.5 h of incubation at 25°C, and the relative growth rate of fungal colonies (mm/d) was calculated. Asterisks indicate statistically significant differences between ∆*hda1* mutants and the WT within each treatment group (*n* ≥ 3, *P < 0.05, **P < 0.01, ***P < 0.001; details of the statistical evaluation are given in Table S2).

Osmotic stress triggered by sorbitol or sodium chloride as well as oxidative stress triggered by menadione or hydrogen peroxide led to a significantly reduced relative growth rate of the *∆hda1* mutants compared to the WT (Fig. 6). In contrast, cell wall stress triggered by 600 µM Congo red and plasma membrane stress triggered by 0.014% sodium dodecyl sulfate did not affect growth of the *∆hda1* mutants (data not shown).

### *Hda1* gene deletion leads to altered metabolite fingerprints

Treatment of liquid *T. atroviride* cultures with the oxidative stress reagents menadione and hydrogen peroxide did not trigger a phenotype in ∆*hda1* on a metabolic level in HPTLC analysis (data not shown). Therefore, osmotic stress treatment with sorbitol was chosen for further analysis. To this end, the ∆*hda1A* mutant and the WT were cultivated in potato dextrose broth (PDB) and PDB supplemented with sorbitol. Under both conditions, the mutant produced significantly less biomass than the WT, while biomass production of both strains was similar between the untreated control (PDB) and the sorbitol treatment (Fig. S3).

**Fig 7:**
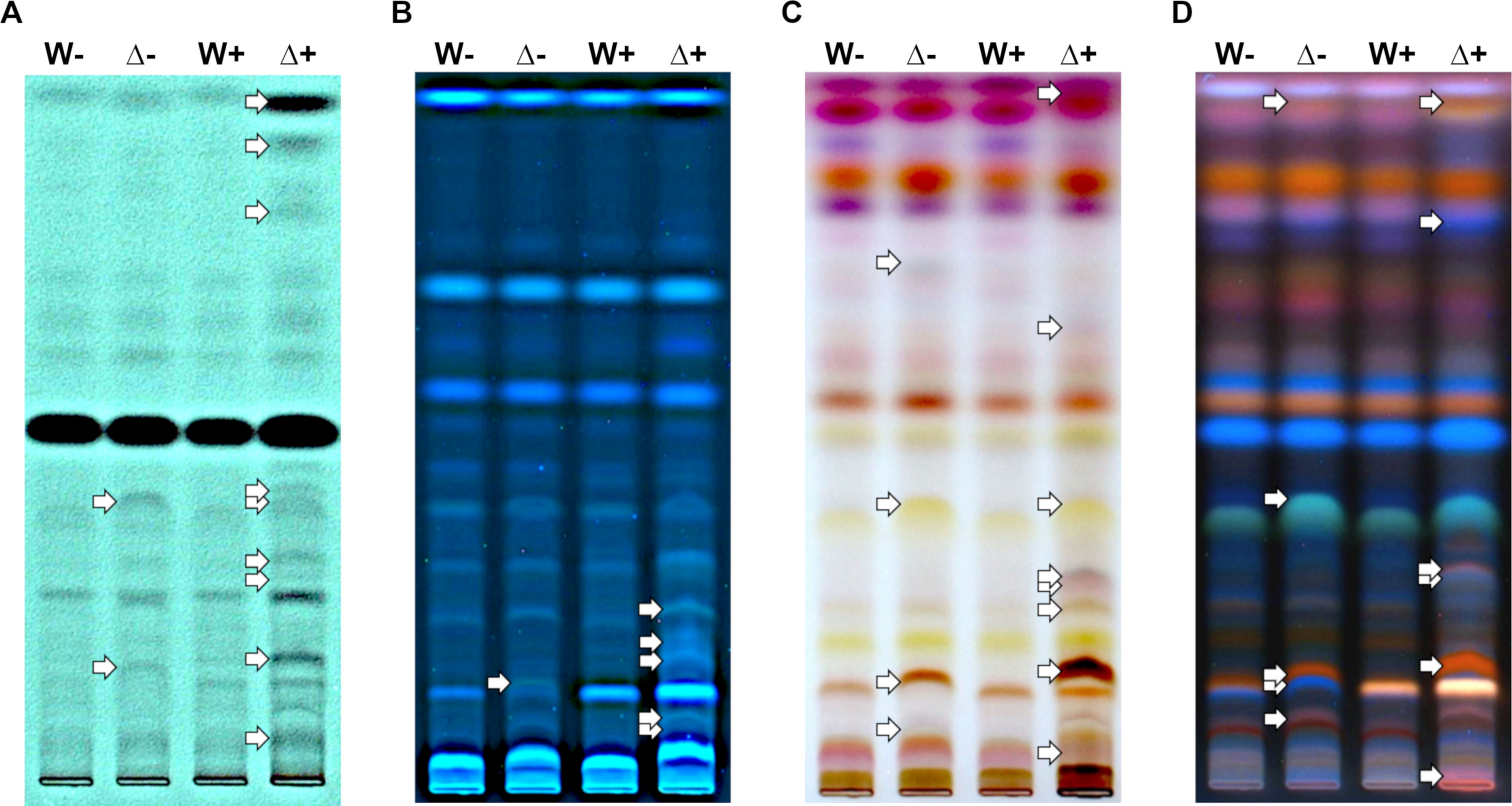
HPTLC fingerprint of metabolites secreted by the ∆*hda1A* mutant and the wild type upon growth in liquid PDB in the presence or absence of sorbitol. Wild type (**W**) and *∆hda1A* (*∆*) were grown in potato dextrose broth in the absence (−) or presence of sorbitol (+). Strains were cultivated at 25°C and 250 rpm for a total time span of 54 h. Sorbitol (50 mM) was added as a single pulse after 30 h of incubation. Metabolites were extracted from culture supernatants and subjected to HPTLC analysis. Photos were taken at 254 nm (remission; UV absorbing substances; **A**), white light (transmission; visible substances; **C**), and 366 nm (remission; fluorescent substances; **B and D**) before (**A and B**) and after (**C and D**) derivatization with p-anisaldehyde sulfuric acid reagent. White arrows highlight differences (bands with higher intensity and additional bands) in the metabolite fingerprints of the *∆hda1A* mutant compared to the WT. The figure shows one representative plate out of four biological replicates (*n* = 4). The additional three replicates are given in Fig. S2.

Comparative metabolite fingerprinting of culture supernatants of the ∆*hda1A* mutant and the WT by HPTLC analysis revealed reproducible differences between the tested strains (Fig. 7; Fig. S4). These differences were subtle upon cultivation in PDB, while a stronger metabolite heterogeneity became visible in sorbitol-treated cultures (Fig. 7A and B). Derivatization with anisaldehyde (Fig. 7C and D)—a universal reagent for the detection of diverse natural products (23)—as well as the visualization of fluorescent compounds with UV_366_ light enabled the detection of additional compounds and improved the comparison of the metabolic fingerprints of the two strains tested. While upon growth in PDB, few bands differed in their intensity or presence between the WT and the ∆*hda1A* mutant, the treatment with sorbitol led to major changes in the metabolite profiles of ∆*hda1*A compared to the WT, as indicated by additional or more intense bands. The WT itself only showed minor alterations in the metabolite profile when treated with sorbitol.

### Loss of Hda1 results in significant gene expression changes

**Fig 8:**
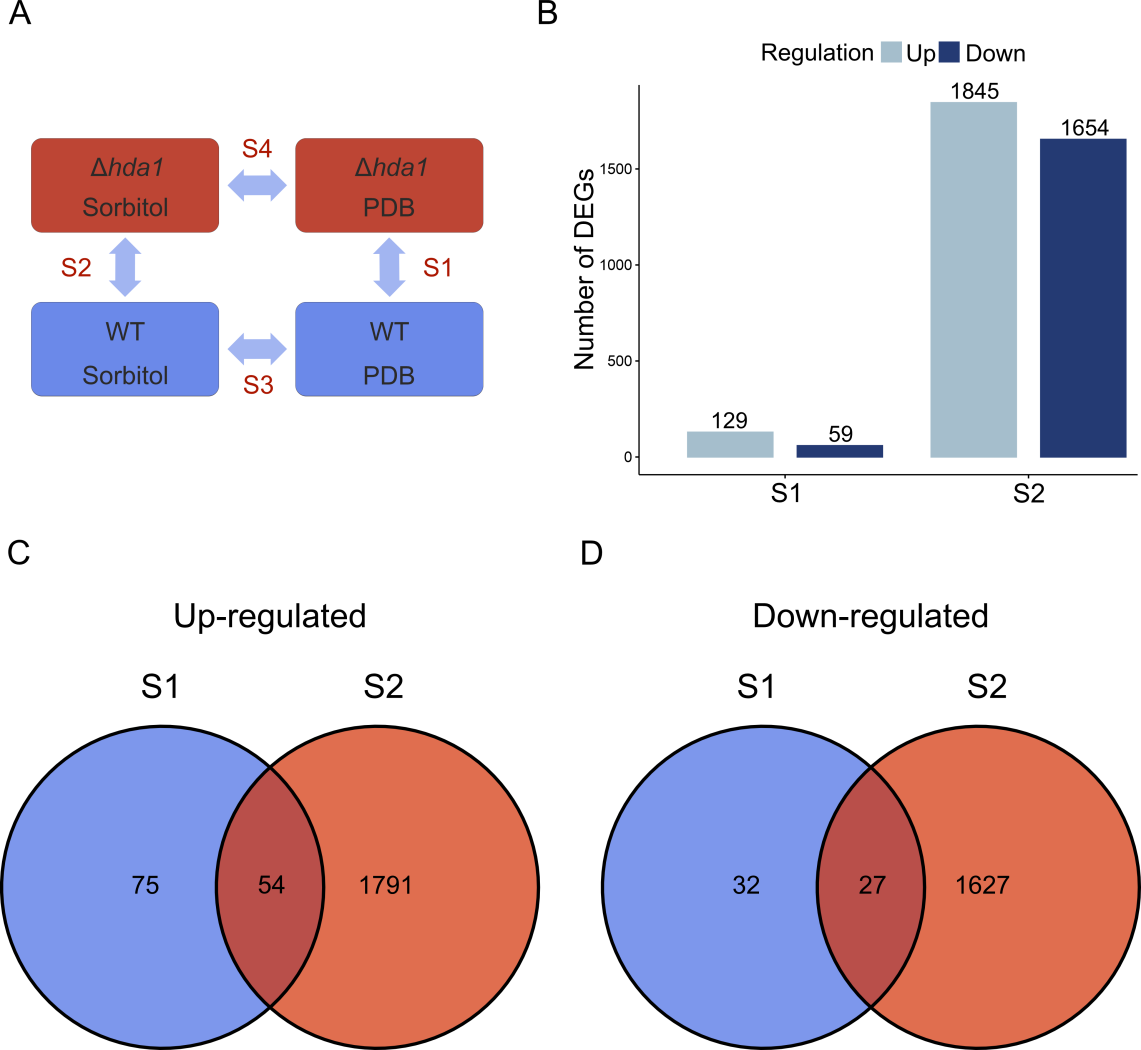
Comparison of differentially expressed genes (DEGs) in the *T. atroviride* ∆*hda1A* mutant *versus* the wild type upon growth in PDB in the absence (**S1**) or presence (**S2**) of sorbitol. (**A**) Scheme of the four computed comparisons for transcriptome analyses: transcriptomes of the ∆*hda1A* mutant versus the wild type upon growth in PDB in the absence (**S1**) or presence (**S2**) of sorbitol. Transcriptomes of the wild type grown in the presence of sorbitol versus grown in untreated PDB (**S3**) and *Δhda1A* mutant grown in the presence of sorbitol versus grown in untreated PDB (**S4**). (B−D) DEGs between the *Δhda1A* mutant versus WT grown in untreated PDB (**S1**) and in PDB in the presence of sorbitol (**S2**). DEGs with adjusted *P*-values ≤0.05 and absolute fold change values of log_2_FC ≥1 were considered either up- or down-regulated. (**B**) Distribution of up- and down-regulated DEGs between comparisons. The number of DEGs is indicated above the bars. Venn diagrams of up-regulated (**C**) and down-regulated (**D**) DEGs of S1 and S2. The non-overlapping regions represent the number of DEGs unique to each comparison. Overlapping regions represent the number of DEGs shared by the comparison groups.

For the comparative analyses of the RNA-seq data of the *Δhda1A* mutant and the WT upon growth in PDB and PDB supplemented with sorbitol, four data sets were generated (Fig. 8A): *Δhda1A* versus WT in PDB (S1), *Δhda1A* versus WT upon sorbitol treatment (S2), WT sorbitol treatment versus PDB (S3), and *Δhda1A* sorbitol treatment versus PDB (S4). Genes with an adjusted *P*-value ≤0.05 and |log_2_FC| >1 were considered as differentially expressed (Supplementary File A).

A total of 188 and 3,499 differentially expressed genes (DEGs), respectively, showed a significantly different regulation in the *Δhda1A* mutant compared to the WT upon growth without (S1) and with (S2) osmotic stress (Fig. 8B; Supplementary File A). Among these DEGs, 75 up- and 32 down-regulated genes were exclusive for the S1 comparison, 54 up- and 27 down-regulated genes were shared between S1 and S2, and 1791 up- and 1627 down-regulated genes were exclusive for S2 (Fig. 8C and D). In S1, we found 12 significantly up-regulated genes linked to mycoparasitism, half of which were peptidases (e.g., aspergillopepsin-2). The other half comprised oxidoreductases, one laccase, one monooxygenase, and one cutinase. The genes encoding a S-adenosyl-methyltransferase and a ferulic acid decarboxylase were among the most up-regulated genes in the S1 data set. Among the most down-regulated genes in S1, candidates involved in conidiation (*agl1*), GPCR trafficking (arrestin-like protein), and alkaloid biosynthesis (catalase) were identified. In addition, nine genes encoding enzymes with reductase activity that showed down-regulation in the mutant (e.g., cyclohexanone monooxygenase) were found.

The DEGs of the sorbitol treatment compared to growth in untreated media of the WT (S3) and Δ*hda1A* (S4) represent the response of the two strains to osmotic stress (Fig. S5). The high number of DEGs exclusively found in the S4 data set (1,856 up-regulated, 1,600 down-regulated) indicates a significant influence of osmotic stress on the *Δhda1A* mutant, while the expression of only a low number of genes was affected by sorbitol in the WT (72 up-regulated, 16 down-regulated).

### Functional classification of DEGs defines the role of Hda1

Gene Ontology (GO) and Kyoto Encyclopedia of Genes and Genomes (KEGG) enrichment analysis of DEGs was performed on the S1 and S2 data sets. The DEGs up-regulated in the ∆*hda1* mutant compared to the WT upon growth in PDB (comparison S1) were mainly associated with the molecular functions “oxidoreductase activity” and “catalytic activity” (Supplementary File B). Notably, down-regulated DEGs in the same comparison were also enriched in “oxidoreductase activity” and genes down-regulated in the mutant linked to “mitochondrial inner membrane.” KEGG analysis highlighted that *hda1* gene deletion mainly affected the pathway categories

"environmental information processing,” “genetic information processing,” and “metabolism” (Supplementary File B). Arachidonic acid metabolism and nitrogen metabolism pathways were the most gene-enriched pathways. Other pathways, including mitogen-activated protein kinase (MAPK) signaling, homologous recombination, and protein processing, were also positively modulated in S1. Down-regulated pathways in the S1 data set were mostly associated with metabolic pathways, including fatty acid biosynthesis and inositol phosphate metabolism.

**Fig 9:**
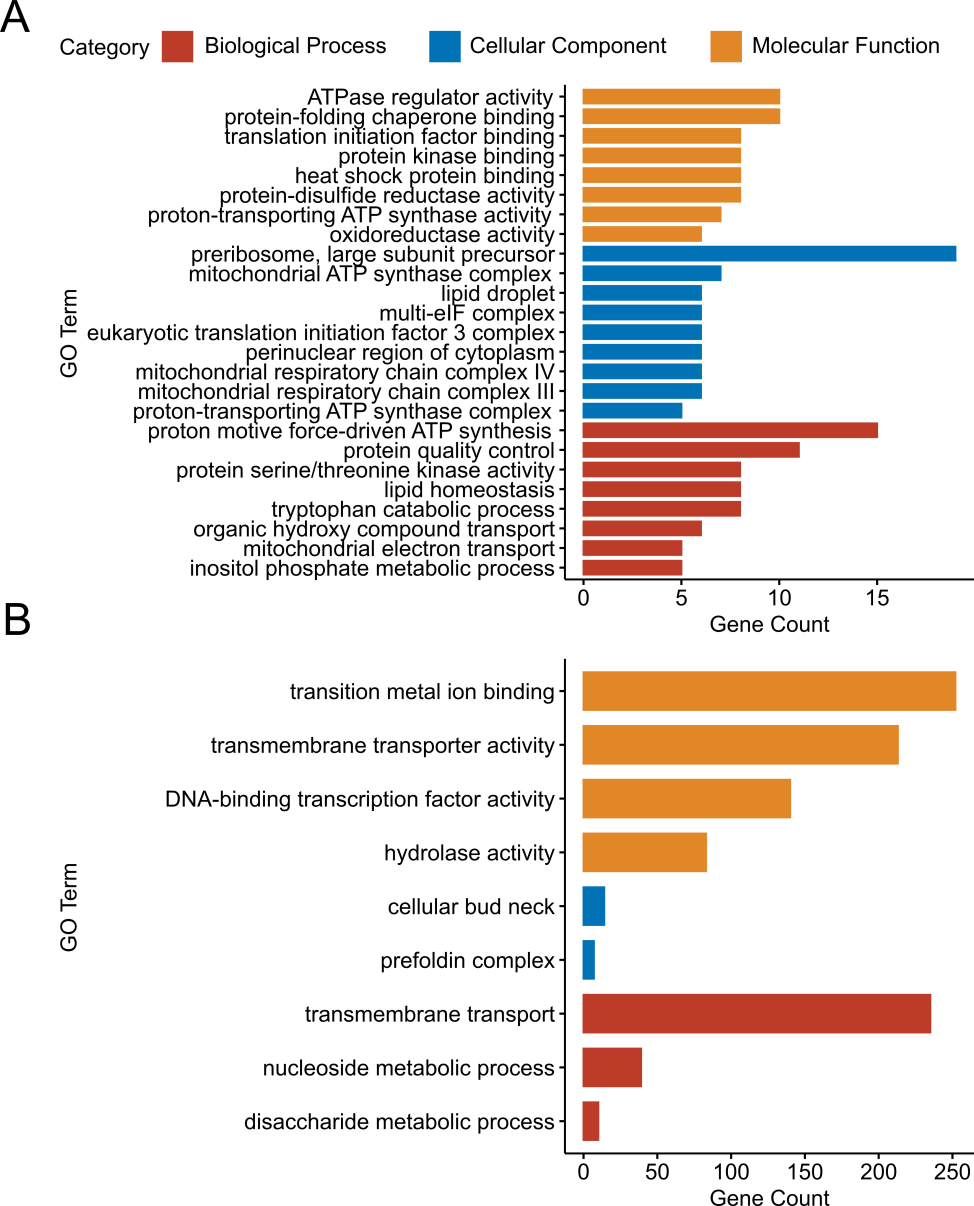
Comparison of the transcriptional responses of the *T. atroviride Δhda1A* mutant and the WT under osmotic stress triggered by sorbitol (S2 comparison). Functional annotation of differentially expressed genes (DEGs; adjusted *P*-values ≤ 0.05) with Gene Ontology (GO). X-axis represents the number of genes assigned to each GO term. GO analysis of DEGs up-regulated (**A**) and down-regulated (**B**) in the *Δhda1A* mutant compared to the WT under osmotic stress.

**Fig 10:**
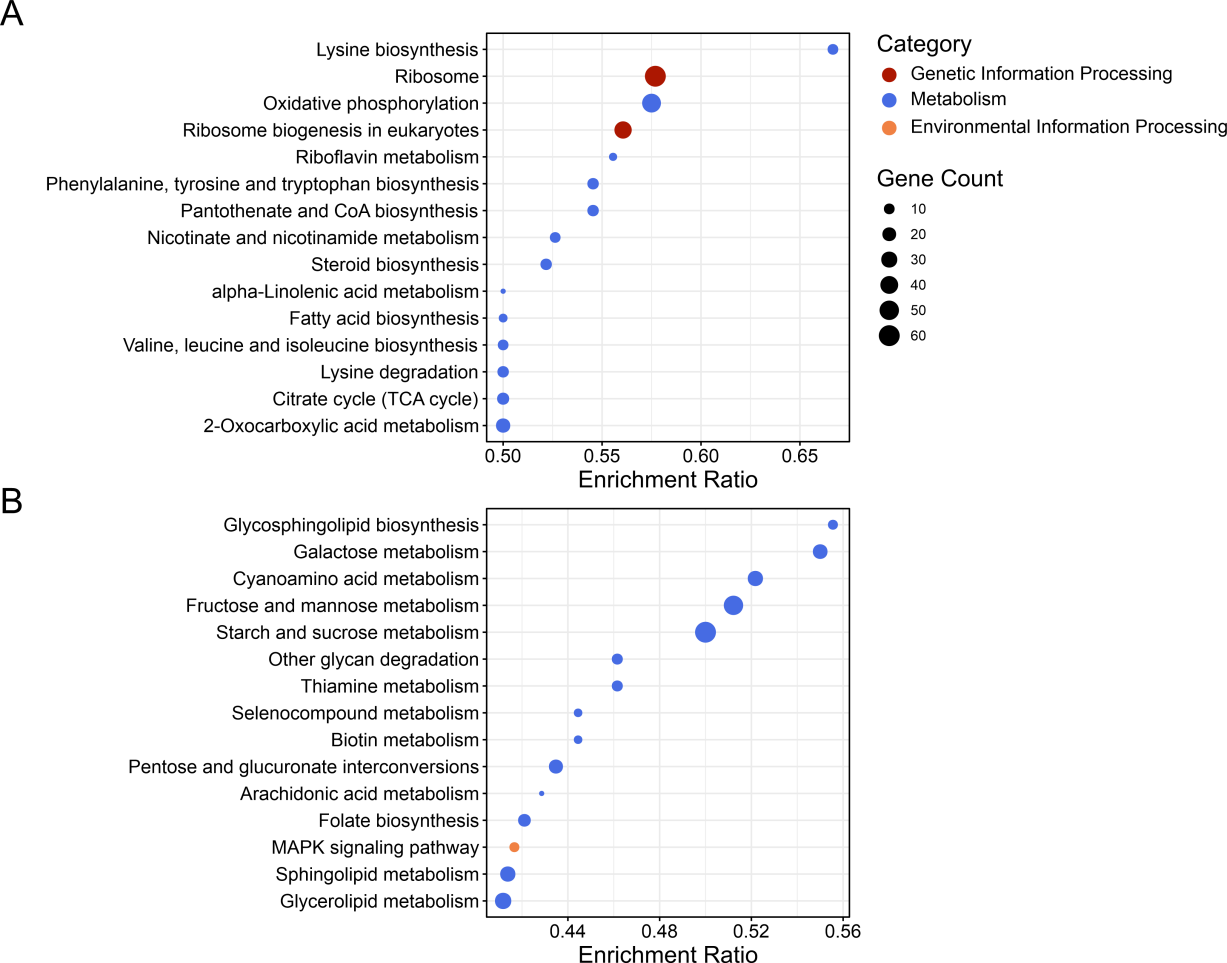
Major pathways differentially regulated in the *T. atroviride Δhda1A* mutant compared to the WT under osmotic stress triggered by sorbitol (comparison S2). Functional annotation of differentially expressed genes (DEGs) with KEGG enrichment analysis. Plots show the 15 most enriched pathways of up- and down-regulated DEGs (adjusted *P*-values ≤0.05). The color of the dots indicates the category of the KEGG term. The sizes of the dots represent the number of enriched genes involved in each row. The x-axis represents the enrichment score. KEGG enrichment analysis of DEGs up-regulated (**A**) and down-regulated (**B**) in the *Δhda1A* mutant compared to the WT under osmotic stress conditions.

GO and KEGG analyses on the DEGs up-regulated in the *∆hda1* mutant compared to the WT upon sorbitol treatment (S2 comparison) revealed a significantly stronger osmotic stress response of the mutant. Enriched terms in the S2 data set indicate an involvement in “signaling” and “transport” (i.e., inositol phosphates, mitochondrial electron transport, and proton-transporting ATP synthase complex), “metabolism” (i.e., oxidoreductase, lysine biosynthesis, and oxidative phosphorylation), and “stress response” (i.e., heat shock protein binding) (Fig. 9A and 10A; Supplementary File C). In the “molecular functions” category, “ATPase regulator activity” was the most enriched GO term, followed by “protein-folding chaperone binding” and “translation initiation factor binding.” In the “cellular component” category, the majority of genes were involved in “pre-ribosome.” Within the “biological process” category, highly represented genes belonged to “response to ATP synthesis,” “protein quality control,” “lipid homeostasis,” and “tryptophan catabolic process.” Deletion of *hda1* had a much stronger effect on gene expression under sorbitol treatment compared to its effect in the untreated conditions. Specifically, 46 DEGs were enriched in oxidative phosphorylation; 22 DEGs, in 2-oxocarboxylic acid metabolism; 14 DEGs, in citrate cycle; and 12 DEGs, in the biosynthesis of pantothenate. Ribosome pathway and biogenesis were also prominently up-regulated. On the other hand, down-regulated genes in the S2 comparison were mainly enriched in the GO terms “transmembrane transport,” “DNA-binding transcription factor activity,” and “hydrolase activity” (Fig. 9B). Down-regulated pathways were mainly involved in “metabolism” (i.e., glycosphingolipid biosynthesis, galactose metabolism, cyanoamino acid metabolism, and starch and sucrose metabolism) and MAPK signaling (Fig. 10B).

### Hda1 governs the expression of secondary metabolism-associated genes

Although no core enzymes were among the significantly up- and down-regulated genes in the S1 data set, six genes located in five different BGCs (two PKS type, two NRPS type, and one TC type) were significantly up-regulated (Table S3). Two genes (encoding oxidoreductase and MFS transporter) are part of the same PKS-type cluster. Beside the genes located in BGCs themselves, transcription factors (TFs) play a crucial role in fungal secondary metabolite (SM) biosynthesis. Eight TF-encoding genes were significantly up-regulated in the *Δhda1A* mutant (Table S4), primarily belonging to the Zn(II)_2_Cys_6_ and C_2_H_2_ zinc finger families, while one down-regulated TF-encoding gene was a member of the bZIP class.

**Fig 11:**
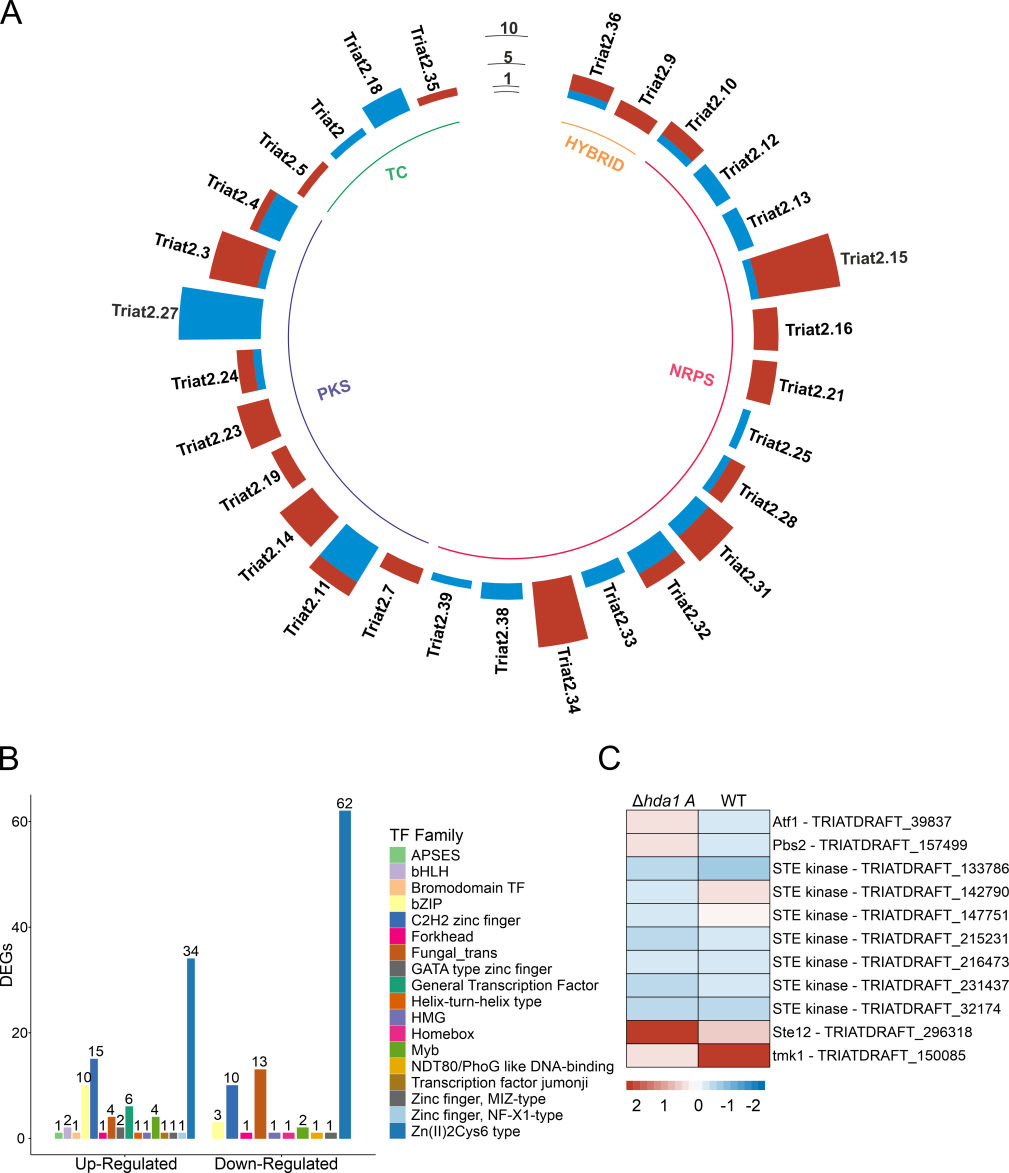
DEGs between the *T. atroviride Δhda1A* mutant versus the WT upon cultivation in the presence of sorbitol (**S2**) that are associated with secondary metabolism, osmotic stress response, and transcription factor activity. DEGs with adjusted *P*-values ≤0.05 and absolute fold change values of log_2_FC ≥1 were considered either up- or down-regulated. (**A**) Number of DEGs found in BCGs, grouped in cluster (Triat2 JGI cluster) and respective cluster types (PKS, NRPS, TC, hybrid). Blue bars indicate the number of up-regulated genes, and red bars, the number of down-regulated genes in the respective cluster. (**B**) Bar chart displaying the number of transcription factors (TFs) detected among the significantly regulated DEGs grouped in up- and down-regulated DEGs. Colors represent the respective TF family. (**C**) Heatmap of selected genes involved in the HOG MAPK signalling pathway. Heatmap shows normalized count with blue to red color key (blue, lower normalized count; red, higher normalized count).

According to the results of GO and KEGG analyses of the S2 comparison, the deletion of *hda1* had a substantial impact on metabolism upon osmotic stress treatment. This effect was also observable at the level of BGCs, with 8 core enzyme-encoding genes and 43 genes located in 19 different BGCs being significantly up-regulated (Fig. 11A; Supplementary File C). The affected BGCs comprise 11 NRPS-type, 5 PKS-type, 2 TC-type, and 1 hybrid clusters. On the other hand, 13 genes coding for core enzymes and, in total, 65 genes located in 23 different BGCs were significantly down-regulated (Fig. 11A; Supplementary File C). Here, the affected BGCs comprise 12 NRPS-type, 8 PKS-type, 2 hybrid, and 1 TC- type clusters. In terms of TFs, 85 were up- and 95 were down-regulated (Fig. 11B; Supplementary File C). The most abundant up-regulated TFs were members of the Zn(II)_2_Cys_6_ class (34 genes), C_2_H_2_ zinc fingers (14 genes), and bZIP (10 genes). Similar to the up-regulated TF candidates, the down-regulated TFs were mainly of the Zn(II)_2_Cys_6_ class (62 genes), fungal specific TFs (13 genes), and C_2_H_2_ zinc fingers (10 genes).

### Loss of Hda1 affects the expression of HOG MAPK pathway components upon osmotic stress

The identified DEGs of the S2 data set were explored for components of the high-osmolarity glycerol (HOG) MAPK pathway (Fig. 11C), which is activated by increased environmental osmolarity. Furthermore, the HOG signaling pathway has been reported to influence the regulation and biosynthesis of secondary metabolites in *T. reesei* (24).

In the *Δhda1*A mutant, the gene for the orthologue of the yeast Pbs2 MAPKK, which acts upstream of the Hog1 MAPK in *S. cerevisiae* (25), was significantly up-regulated compared to the WT upon sorbitol treatment. Interestingly, neither *tmk3* [encoding the ortholog of yeast HOG1 (26)] nor *tmk2* [encoding the ortholog of yeast cell wall integrity MAPK Slt2 (26)] were differentially expressed in the S2 data set. On the other hand, *tmk1* (coding for the ortholog of the yeast pheromone response pathway MAPK Fus3) was significantly down-regulated in the S2 comparison, while the gene for the transcriptional regulator Ste12, which mediates outcomes of the Tmk1 MAPK pathway (27), was significantly up-regulated. Furthermore, an orthologue of yeast Atf1 (28), which functions as an essential downstream component of the Hog1 MAPK pathway in *S. cerevisiae*, was significantly up-regulated in the S2 data set. In filamentous fungi, Atf1 is involved in multiple cellular processes and regulates the transcription of genes related to stress response and secondary metabolism (28).

### Hda1 impacts the chemical diversity of *T. atroviride* under osmotic stress

HPTLC analysis pointed to a regulatory role of Hda1 in the biosynthesis of secreted metabolites in *T. atroviride* which was further triggered by sorbitol treatment. Putative metabolites were hence linked to each differentially expressed BGC of the S2 data set (Table 1) by using antiSMASH v5.0.

**TABLE 1:** BGCs differentially regulated in the *T. atroviride Δhda1A* mutant compared to the WT under osmotic stress conditions (S2) and their predicted putative metabolites^*a*^

JGI cluster	Cluster type	Most similar known cluster	Similarity (%)	Regulation
Triat2.27	PKS	Ascochlorin	50	Up
Triat2.3	PKS	6-Methylsalicylic acid	27	Up
Triat2	TC	Clavaric acid	100	Up
Triat2.14	PKS	Ankaflavin	16	Down
Triat2.21	NRPS	Enniatin	100	Down
Triat2.34	NRPS	Fusaric acid	45	Down
Triat2.5	PKS	Trichoxide	50	Down

^*a*^ DEGs with adjusted *P*-values ≤0.05 and absolute fold change values of log_2_FC ≥1 were considered either up- or down-regulated. Most similar known secondary metabolite cluster in the *T. atroviride* genome was identified by antiSMASH.

Two differentially expressed BGCs of the S2 data set showed sequence identity to clusters putatively involved in the biosynthesis of clavaric acid (up-regulated) and enniatin (down-regulated). Further up-regulated BGCs in the *∆hda1* mutant compared to the WT upon sorbitol treatment match to similar clusters responsible for the biosynthesis of ascochlorin and 6-methylsalicylic acid. Additional down-regulated BGCs in the S2 data set match with a similar cluster of *T. virens* responsible for the biosynthesis of the antifungal substance trichoxide (29) and a *Fusarium verticilloides* BGC for fusaric acid biosynthesis (30).

## DISCUSSION

Sparse information is available on the functional role of HDACs in mycoparasitic fungi. Solely Hda-2, the orthologue of the class I HDAC HOS2 of *S. cerevisiae*, has been functionally characterized in *T. atroviride* to date and is involved in the regulation of growth, conidiation, blue-light perception, oxidative stress response (16), and VOC biosynthesis and in plant defense response against foliar fungal pathogens (31).

Since mycoparasitic interaction assays are traditionally inoculated with mycelia-covered plugs, we compared this inoculation type to conidial inoculation. As described for *F. graminearum* (32), *A. alternata* (33), and *F. fujikuroi* (21), plug-inoculated *∆hda1* cultures of *T. atroviride* exhibited a similar phenotype and growth rate as the WT. In contrast, the *∆hda1* mutants showed significantly reduced growth in spore-inoculated solid cultures and in liquid cultures—a phenotype which was not reasoned by a conidial germination defect. These findings are comparable with *M. oryzae* (34), where deletion of the *hda1* gene also reduced growth in surface and liquid cultivation. In *C. albicans*, *hdaA* deletion decreased growth in drop-plate cultures (35), and in *A. fumigatus*, it led not only to a reduced radial growth of spore-inoculated cultures but also to a delay in germination (19). In contrast to our findings, ∆*hdaA* mutants of *A. flavus* (36), *A. nidulans* (17), and *C. fulvum* (37) were not affected in growth and/or germination under standard cultivation conditions.

*Trichoderma* VOCs were described to affect the interaction with plants as well as the mycoparasitic interaction with host fungi (38–41). A recent study demonstrated an influence of *hda-2* gene deletion in *T. atroviride* on the quantity of emitted VOCs and the induction of systemic resistance and plant growth promotion in *A. thaliana* (31). Similar to these results, we found the quantity of four VOCs, i.e., 2-methy-butanol, 3-methyl-butanol, 3-octanone, and ethanol, affected in their emission to the culture headspace in the ∆*hda1* mutant. Three of these VOC—which were all described as antifungal agents (42–46)—were emitted at higher levels by the *∆hda1* mutant, while 3-octanone was found in lower quantities in the headspace. The inhibitory effect of secreted metabolites of *T. atroviride* on *B. cinerea* germination and growth was significantly enhanced in *∆hda1*, indicating higher amounts of inhibitory, antifungal metabolites secreted by *∆hda1* cultures. Indeed, genes in the BGC responsible for the biosynthesis of 6-methylsalicalic acid, a phytotoxic compound that was shown to inhibit the growth of *B. cinerea* (47), were up-regulated in the ∆*hda1* mutant. In addition, several genes coding for peptidase, hydrolase, and oxidoreductase were up-regulated upon deletion of *hda1*, and these play a key role in *Trichoderma* mycoparasitism (48). In contrast to these findings, *hda1* gene deletion leads to a clear reduction of the mycoparasitic activity of *T. atroviride* in direct confrontation with host fungi. The mycoparasitic overgrowth of *R. solani* by the *∆hda1* mutant was severely delayed, whereas *B. cinerea* could not be completely overgrown. To date, the majority of studies reported on a critical impact of the class I HDAC Hos2 on virulence or pathogenicity in other fungi (10, 11), and, consistent with our findings, the class II HDAC Hda1 was also described as virulence determinant in some species. In *Cryptococcus neoformans*, Hda1 is a central mediator of virulence (49); in the plant pathogen *M. oryzae*, *hda1* gene deletion caused a 60% reduction of lesions (50); and in *F. fujikuroi*, a 25% reduction of internode elongation in the host plant *Oryza sativa* was reported (21). Based on our results, we conclude that Hda1 plays a host-dependent role in the mycoparasitic interaction of *T. atroviride* and is a determinant for the mycoparasitic success. Since the only primarily occurring delay in spore germination of the *∆hda1* mutants can be neglected in the applied experimental setups, the observed effects may be mainly based on cumulative and alterative effects of Hda1 loss on the global metabolome, which seemed to weaken the mycoparasitic abilities, but at the same time enhance the antifungal activity of the secreted metabolite cocktail.

HDACs are well known to differentially regulate the response to biotic and abiotic stress as well as the biosynthesis of various metabolites in fungi (9, 10, 16, 51–54). Hda1, in particular, was described to affect the oxidative stress response in *S. cerevisiae* (55–57), *A. nidulans* (17, 18), and *P. chrysogenum* (58) and drug resistance and antifungal susceptibility of *S. cerevisiae* (59) and *C. albicans* (35) as well as to differentially and globally govern the biosynthesis of several metabolites in *A. fumigatus* (19), *F. fujikuroi* (21, 60), *M. oryzae* (20), and other fungi (14). In *T. atroviride*, *hda1* deletion also leads to a significantly increased sensitivity to menadione and hydrogen peroxide, triggering oxidative stress upon growth on solid media. In contrast to our findings, in *A. fumigatus* (19), *A. flavus* (36), and *A. thaliana* (33), HdaA/1 was reported as negligible for growth in response to oxidative stress. Furthermore, the *T. atroviride ∆hda1* mutant produced higher quantities of certain substances as well as new metabolites, as indicated by HPTLC analysis of liquid culture supernatants. Our transcriptomic analysis suggests that Hda1 is one of the master regulators of secondary metabolism, affecting several biosynthetic and metabolic pathways in *T. atroviride*, thereby being involved in both repression and induction of certain clusters. Similar findings have been reported for *A. fumigatus* (19) and *F. fujikuroi* (21). To our surprise, we obtained evidence that Hda1 is also involved in the response to osmotic stress in *T. atroviride*. The *∆hda1* mutants showed a significantly decreased growth on sorbitol- and sodium chloride-supplemented solid media. This osmosensitive phenotype was also represented on a metabolic level, since sorbitol further extensively triggered the enhanced and additional secretion of metabolites in the *∆hda1* mutant in liquid culture. In addition, the ∆*hda1* mutant exhibited a strong alteration in its transcriptional landscape under sorbitol treatment, highlighting the pronounced sensitivity to osmotic stress, whereas the WT only showed a minor response to osmotic stress. Accordingly, transcription of BGCs in the *∆hda1* mutant was heavily affected under these conditions. Among the up-regulated BGCs were those related to clavaric acid, a triterpenoidal inhibitor with antitumor and anti-oncogenic activities (61), and ascochlorin, an antibiotic first isolated from the phytopathogenic fungus *Ascochyta viciae* (62). To date, ascochlorin has not yet been identified or characterized in *Trichoderma* species, whereas various ascochlorin analogues have been isolated from multiple fungi within the Hypocreales order (63). Osmotic stress in filamentous fungi is processed by the HOG MAPK cascade (64). Under osmotic stress, yeast Pbs2 activates the Hog1 MAPK, which induces a set of osmoadaptive responses (65). In the *T. atroviride ∆hda1* mutant, the yeast Pbs2 orthologue was significantly up-regulated upon sorbitol treatment, implying that hda1 gene deletion affects the regulation of HOG signaling. In yeast, the HOG pathway controls gene expression through several mechanism, including chromatin modification via recruitment of the Rpd3 histone deacetylase complex. Deletion of the Rpd3 histone deacetylase encoding gene leads to osmosensitive cells (66). Reports of the role of Hda1 in osmotic stress response to date are controversial. Similar to our findings, sodium chloride treatment resulted in reduced growth of *A. alternata ∆hda1* strains on solid media, which was not the case upon the addition of sorbitol (33). In contrast, *A. oryzae ∆hdaA* mutants displayed a unique osmosensitive phenotype in liquid but not on solid media (67). Osmotic stress did not affect growth of *A. flavus ∆hdaA* mutants (36), which is in accordance with reports from yeast but in contrast to our findings in *T. atroviride*, since no influence (66) or even a higher resistance (68) to osmotic stress was reported for the ∆*hda1* strain of *S. cerevisiae*.

Based on our results, we conclude that Hda1 affects the response of *T. atroviride* to oxidative and osmotic stress as well as the biosynthesis of several metabolites, the latter being further triggered upon osmotic stress treatment.

## MATERIALS AND METHODS

### Strains and cultivation conditions

The mycoparasite *Trichoderma atroviride* P1 (ATCC 74058; Ascomycota), its deletion mutants ∆*hda1*A, ∆*hda1*B, and ∆*hda1*C and the plant-pathogenic host fungi *Rhizoctonia solani* (Basidiomycota; pathogenic isolate obtained from the collection of the Institute of Plant Pathology, Università degli Studi di Napoli “Federico II,” Naples, Italy) and *Botrytis cinerea* B05.10 (Ascomycota) were applied in this study.

Surface cultures were grown on potato dextrose agar (PDA; Becton, Dickinson and Company, Le Pont De Claix, France) and liquid cultures in PDB (Becton, Dickinson and Company, Le Pont De Claix, France). A concentration of 200 µg/mL hygromycin B (Calbiochem, Merck KGaA, Darmstadt, Germany) was used for mutant selection. If not stated otherwise, four biological replicates of the WT and the three *hda1* deletion mutants (∆*hda1*A, ∆*hda1*B, and ∆*hda1*C) were processed in all phenotypic characterization experiments. Since all three deletion mutants showed reproducible and comparable phenotypes and characteristics in the growth and confrontation assays, *∆hda1A* was randomly selected for detailed studies at the metabolic and transcriptomic levels.

To guarantee standardized growth, fungi were pre-cultured by passaging a 6-mm diameter agar plug of the actively growing colony margin of the fungal colony after 2 days. This procedure was repeated two times after 1.5 days each. The mycelia-covered side of the agar plug was placed upside down onto a fresh agar plate to reach exponential growth at 25°C under light-dark conditions (12:12 h cycle, 300 Lux; Snijders Micro Clima-Series TM Labs Economic Lux Chamber; Snijders Labs, Tiburg, Netherlands). The radial growth rates were determined from cultures developing from exponentially growing mycelia as well as from fresh spores (1 * 10^5^ spores/2 µL dot). The colony diameters were measured after 48 h of growth, and the radial growth rate (mm/d) was calculated.

For cultivation in liquid media, 5 * 10^6^ spores/mL were inoculated into 50 mL of PDB in 250-mL Erlenmeyer flasks and incubated at 25°C and 250 rpm for a total of 54 h. After 30 h of growth, PDB was supplemented with a single pulse of 50 mM sorbitol as osmotic stress treatment, and fungi were grown for additional 24 h. The dry weight (DW) was determined from five biological replicates, and the biomass production was calculated (g DW/L). For RNA extraction, protein extraction, and metabolite profiling, the mycelial biomass and the supernatants of four biological replicates were harvested by vacuum filtration. The mycelial biomass was immediately frozen in liquid nitrogen and stored at −80°C. The supernatants were stored at −20°C. The assay for determination of germination was done in three biological replicates with 5 * 10^6^ spores/ mL in 50-mL PDB in 250-mL Erlenmeyer flasks according to (69). The germination rate (%) was counted and calculated every hour between 7 and 13 h of growth at 25°C and 250 rpm.

### Bioinformatic analysis and generation of deletion mutants

The *T. atroviride* Hda1 orthologue (ID 386002; https://mycocosm.jgi.doe.gov/Triatrov1/Triatrov1.home.html) was identified via BLASTp analysis using the functionally characterized proteins of *Fusarium fujikuroi* FfHda1 (XP_023435500.1), *Magnaporthe oryzae* HDA1 (XP_003717862.1), *Alternaria alternata* Hda1 (RYN77957.1), *Aspergillus nidulans* HdaA (XP_050467238.1), *Cladosporium fulvum* HdaA (Clafu1_192224), *Saccharomyces cerevisiae* Hda1 (NP_014377.1), and *Aspergillus fumigatus* HdaA (XP_748144.1) (Table S1). The phylogenetic tree (Fig. S1) was constructed with the maximum likelihood method of MEGA 11 (70) using 500 bootstrap replications based on the complete protein sequence of *T. atroviride* protein ID 386002 and known HDAC sequences, i.e., *A. alternata* Hda1 (RYN77957.1), *A. fumigatus* HdaA (XP_748144.1), HosA (XP_749513.1), HosB (XP_746614.1), RpdA (XP_749474.1), *B. cinerea* Hda1 (XP_024553282.1), Hos2 (XP_001550994.1), Rpd3 (XP_001560049.1), *Candida albicans* Hda1 (XP_718271.1), Hos2 (XP_717754.2), Hos3 (XP_720522.2), *C. fulvum* HdaA (Clafu1_192224), *F. fujikuroi* FfHda1 (XP_023435500.1), FhHda2 (XP_023424030.1), *Neurospora crassa* Hda-1 (XP_956974.3), *S. cerevisiae* Hda1 (NP_014377.1), Hos2 (NP_011321.1), Hos3 (NP_015209.1), Rpd3 (NP_014069.1), *T. atroviride* Hda-2 (Triatrov_225831), and *Ustilago maydis* Hda1 (AAM15960.1). *T. atroviride* and *C. fulvum* protein sequences were retrieved from Joint Genome Institute (JGI). All other protein sequences were retrieved from National Center for Biotechnology Information (NCBI).

For the generation of *hda1* deletion mutants, the following fragments were PCR amplified and assembled with NEBuilder HiFi DNA Assembly Kit (New, England Biolabs, Germany): (i) a 1-kb region upstream of the *hda1* coding sequence (amplified with primers hda1_5KO-F1 + hda1_5KO-R1), (ii) a 1-kb region downstream of the *hda1* coding sequence (amplified with the primers hda1_3KO-F1 + hda1_3KO-R1), (iii) the hygromycin resistance conferring selection cassette containing the *hph* gene (71) (amplified with primers hph-pLS3-CRIBc-F1 + hph-pLS3-CRIBc-R1 and fused with the primers hda1_39952-hph-F1 + hda1_39952-hph_R1), and (iv) the backbone from pLS3-CRIBc-mBasicGFP (72) (amplified with primers pRITA-bb-F1 and pRITA-bb-R1). The selection cassette was split into a 5′ (amplified with primers hda1_39952-HY-F1 + Catlett HY-R) and 3′ (amplified with primers Catlett YG-R + hda1_39952-YG-R1) split marker fragment for homologous recombination via the split marker approach (73) and transformed into *T. atroviride* protoplasts as described previously (74). All primer sequences are given in Table S3. The resulting transformants were purified to mitotic stability by three rounds of single spore isolation on media containing 200 µg/mL hygromycin B (Calbiochem, Merck KGaA, Darmstadt, Germany).

Three independent and mitotically stable deletion mutants, named ∆*hda1*A, ∆*hda1*B, and ∆*hda1*C, were verified via genotyping PCR (Fig. S2B). The locus-specific integration of the deletion cassette was proven by using the two *hph*- and locus-specific primer pairings hl1 and hl2 (using primers hda1_39952_C_F1 + Pgapdh_hph_RT_R1 and Pgapdh_hph_RT_F1 + hda1_39952_C_R1). The successful deletion and hence absence of the *hda1* target locus was verified with the two *hda1*- and locus-specific primer pairings gl1 and gl2 (using primers hda1_39952_C_F1 + hda1_39952_RT_F1 and hda1_39952_RT_R1 + hda1_39952_C_R1). The gene *sar1* (75) was targeted as internal control (primers 301975_hkc_F_Brunner2008 + 301975_hkc_R_Brunner2008). All primer sequences are given in Table S5. The PCR-based genotyping strategy and the results of the genotyping of ∆*hda1*A, ∆*hda1*B, and ∆*hda1*C are given in Fig. S2A.

### Confrontation assay

To determine the effects of *hda1* gene deletion on the mycoparasitic activity of *T. atroviride*, dual culture plate assays (76) with *R. solani* and *B. cinerea* as host fungi were performed. The mycelia covered surface of 6-mm diameter agar plugs of the final pre-cultures of the WT and the deletion mutants were placed upside down onto the outer edge of a fresh PDA plate, opposing the host fungus. Additionally, each strain was grown alone and in self-confrontation (data not shown). The progress of the mycoparasitic interaction was documented photographically after 5, 6, and 15 days of incubation.

### Inhibition assay

To determine the inhibiting effect of soluble, diffusible metabolites secreted by the *T. atroviride* WT and its *hda1* deletion mutants, inhibition assays with *B. cinerea* as test organism were performed according to reference (76) with slight modifications. A mycelia covered surface of a 6-mm diameter agar plug of the final pre-cultures of the WT and the deletion mutants was placed upside down onto the center of a fresh cellophane-covered PDA plate. The plates were incubated for 32.5 h before the mycelial diameters were measured, and the cellophane covered with the *T. atroviride* mycelia was removed. One hundred microliters of a *B. cinerea* spore suspension with 1 * 10^6^ spores/mL were spread on each plate using sterile 3-mm diameter glass beads. The plates were further incubated for 44.5 h at 25°C in a 12-h/12-h light-dark cycle. The diameters of the inhibition zones in the developing *B. cinerea* cultures were measured, and the inhibition index (%) was calculated as a quotient of the diameter of the zone of *B. cinerea* growth inhibition and the diameter of former *T. atroviride* colony growth.

### VOC analysis

To determine the effect of *hda1* gene deletion on the emission of VOCs by *T. atroviride*, the culture headspace was directly analyzed by GC-IMS (Leibniz University of Hannover, Germany). Measurement parameters are described in reference (77). A 6-mm diameter agar plug from the final pre-cultures of the WT and the deletion mutant ∆*hda1*A was placed upside down to the outmost edge of a sterile 150-mL Schott bottle (Duran GmbH, Mainz, Germany) caped gastight using a Teflon cap (Bohlender GmbH, Grünsfeld, Germany) with gas inlet and outlet and filled with 25-mL PDA and incubated at 25°C. During sampling and headspace measurement, the axenic cultures were illuminated twice a day for 2 h with a LED lamp (LED Lumistixx, OSRAM, Germany) and aerated with 5 mL/min continuous streaming purified air per flask. The emission of VOCs was recorded at 21, 24.5, 43.5, 48.5, 68.5, 72.5, 91.5, 96.5, and 115.5 h of incubation as described earlier (77).

### Stress assay

To determine the effect of *hda1* gene deletion on the response of *T. atroviride* to oxidative, osmotic, and plasma membrane stress, stress assays were performed. Twenty-five-milliliter PDA plates were supplemented with 700 mM sorbitol or 500 mM sodium chloride (NaCl) for inducing osmotic stress and with 2.5 mM hydrogen peroxide (H_2_O_2_) or 50 µM menadione for inducing oxidative stress. Fresh spores (1 * 10^5^ spores/2 µL) were point inoculated onto the center of each plate. Colony diameters were measured after 47.8 h of incubation at 25°C, and the radial growth rate (mm/d) was calculated.

### Metabolite extraction and HPTLC analysis

To screen for alterations in the metabolite profile of ∆*hda1A* compared to the WT, metabolites were extracted from the supernatants of untreated (PDB; control) and sorbitol treated liquid cultures and subjected to HPTLC. An optimized extraction method of our previously described protocol (69) was applied for metabolite extraction from liquid cultures. Liquid cultures of the WT and the ∆*hda1A* mutant were obtained like described above. Six milliliters of the culture supernatants were aliquoted and mixed with 1.1 mL of acetone p.a. (CarlRoth GmbH + Co KG, Karlsruhe, Germany) each. The mixture was incubated for 15 min at room temperature in the ultrasonic bath. After addition of 4.5-mL ethyl acetate p.a. (EtOAc; CarlRoth GmbH + Co KG, Karlsruhe, Germany), the samples were mixed well. Phase separation was obtained by centrifugation at 3,000 × *g* for 1 min, and the upper phase was transferred to a broad glass vial. The EtOAc extraction step was repeated a second time, and the EtOAc extracts were evaporated overnight. The next day, evaporated extracts were re-collected in 140 µL of methanol p.a. (Merck KGaA, Darmstadt, Germany). Chromatographic separation was done on silica gel plates (HPTLC silica gel 60 F254S, Merck KGaA, Darmstadt, Germany) via HPTLC as previously described (69).

### RNA extraction and transcriptome analysis by RNA sequencing

For the analysis of gene expression differences between the WT and ∆*hda1A*, RNA was extracted from the fungal biomass derived from liquid cultures grown in either untreated media (PDB; control) or media pulsed for the final 24 h of cultivation with 50 mM sorbitol as described above. RNA was extracted with the RNeasy Plant Mini Kit (Qiagen, Venlo, Netherlands) according to the manufacturer’s manual. Samples were dissolved in 10 mM TRIS-HCl (Invitrogen, ThermoFisher Scientific Baltic UAB, Vilnius, Lithuania), pH 7, including 1 U/µL RiboLock RNase Inhibitor (ThermoFisher Scientific Baltic UAB, Vilnius, Lithuania). A 2.5-µg RNA was digested with DNAseI (ThermoFisher Scientific Baltic UAB, Vilnius, Lithuania) according to the manufacturer’s protocol. The samples were stored at −80°C and sent to Microsynth AG (Balgach, Switzerland) for Illumina stranded TruSeq RNA library preparation [including poly(A) enrichment], Illumina Sequencing (30 Mio reads, 1*75 bp), and bioinformatic analysis. The reads were mapped to the reference genome of *T. atroviride* IMI 206040 (assembly TRIAT v2.0) by bowtie2 (v2.4.2) in local mapping mode with very sensitive pre-settings. To count the uniquely mapped reads to annotated genes, the software HTSeq-count (v0.11.2) was used. Subsequent normalization of the raw counts and differential gene expression analysis were carried out with help of the R software package DESeq2 (v1.22.2).

### Differential gene expression analysis

Four sets of DEGs were generated, comparing the transcriptomes of Δ*hda1A* versus the WT grown in PDB (S1), Δ*hda1A* versus the WT grown in PDB supplemented with sorbitol (S2), the WT grown in PDB with sorbitol versus grown in untreated PDB (S3), and Δ*hda1A* grown in PDB with sorbitol versus grown in untreated PDB (S4) (Fig. 8A).

The DEGs of each comparison were delimited based on an adjusted *P*-value ≤0.05 and an absolute log_2_ fold change (log_2_FC) value ≥1 for up-regulated genes and ≤−1 for down-regulated genes. Venn diagrams comparing the four sets of DEGs were plotted using the “ggvenn” v0.1.10 R package (78). MAPK-related DEGs were visualized as heatmap generated with the R package “pheatmap” v1.0.12 (79).

### Transcriptome functional annotation

DEGs were associated with their gene IDs in the reference genome, and the sequences were annotated with Blast2GO v6.0.3 (80) to determine the related GO terms. The assembled transcripts were aligned using BLASTx against the NCBI nr database. A BLAST expectation value of 1 * 10^−3^ was applied, and for each target sequence, 25 alignments were made. A sequence mapping was conducted, followed by functional annotation in levels of Gene Ontology Biological Process, Molecular Function, and Cellular Component. Sequences that were not annotated by GO were identified by InterPro (81) and UniProt (82) databases. Fisher enrichment tests for up-regulated (log_2_FC >0) and down-regulated (log_2_FC <0) DEGs were performed with Blast2GO to search for significant differences (false discovery rate [FDR] ≤0.05) in frequencies of GO terms compared to *T. atroviride* IMI 206040. Results of Fisher enrichment test were slimmed in REVIGO (83). The R package “ggpubr” v0.5.0 (84) was used to visualize the enriched GO terms as bar plots. “Gene count” equals the number of genes assigned to a GO term. Metabolic pathway analysis was performed using the KEGG (85) and KOBAS v3.0 (86). We used *T. reesei* as a reference for the KEGG pathway significant enrichment analysis because there was no information for *T. atroviride* available in the KEGG database. The 15 most enriched pathways were visualized as dot plots using “ggplot2” v3.4.1 (87). Secondary metabolite biosynthesis gene clusters were mined using the antiSMASH v6.0 (88) platform.

### Histone extraction and determination of histone acetylation

*T. atroviride* histone proteins were extracted as previously described (89) with slight modification. Briefly, frozen lyophilized mycelia (0.1 g) were ground to powder and solubilized in 5 mL of homogenization buffer (10 mM 1,4-piperazinediethanesulfonic acid, pH 6.9, 5 mM CaCl, 5 mM MgSO_4_, 0.5 M sucrose, 1 mM phenylmethylsulfonyl fluoride, and 10 mM β-mercaptoethanol) containing 300 nM trichostatin A and protease inhibitors. After homogenization and centrifugation steps according to the protocol, basic proteins were extracted overnight with hydrochloric acid. Core histones from the supernatant were then further precipitated with acetone and finally resuspended in 50 µL of 1× Laemmli buffer.

Proteins were separated in 16% polyacrylamide gels and blotted onto a nitrocellulose membrane as previously described (90). Immunological detection was performed with the following antibodies (Merck, Darmstadt, Germany) according to the manufacturer’s instruction: anti-acetyl-histone H3, anti-histone H3 pan, and anti-acetyl-histone H4.

### Graphics processing and statistical data analysis

Plots were generated using the R package “ggpubr” v0.5.0 (84). Digital image processing and labeling of photographs and gel and HPTLC pictures were done with GIMP v2.10.32, Corel Draw 2020, and Adobe Illustrator Artwork 16.0. Statistical analysis of data was performed in GraphPad Prism v5 and v9.4.1. Details of statistical analysis are given in Table S2.

## Data Availability

The transcriptome data set of all the samples has been deposited in the NCBI BioProject database (BioProject number PRJNA1018730).

## SUPPLEMENTAL MATERIAL

Supplemental figures and tables spectrum.03097-23-s0001.pdf Fig. S1 to S5; Tables S1 to S5.

Supplemental File A spectrum.03097-23-s0002.xlsx List of DEGs of the four comparisons S1, S2, S3, and S4.

Supplemental File B spectrum.03097-23-s0003.xlsx GO and KEGG analyses of DEGs between the Δhda1A mutant versus the WT upon cultivation in PDB (S1).

Supplemental File C spectrum.03097-23-s0004.xlsx GO and KEGG analysis of DEGs between the Δhda1A mutant versus the WT upon sorbitol treatment (S2).

ASM does not own the copyrights to Supplemental Material that may be linked to, or accessed through, an article. The authors have granted ASM a non-exclusive, world-wide license to publish the Supplemental Material files. Please contact the corresponding author directly for reuse.

## References

[1] López-Bucio J, Pelagio-Flores R, Herrera-Estrella A. 2015. Trichoderma as biostimulant: exploiting the multilevel properties of a plant beneficial fungus. Sci Hortic 196:109–123. doi:10.1016/j.scienta.2015.08.043

[2] Karlsson M, Atanasova L, Jensen DF, Zeilinger S. 2017. Necrotrophic mycoparasites and their genomes. Microbiol Spectr 5. doi:10.1128/microbiolspec.FUNK-0016-2016 PMC11687461 28281442

[3] Kubicek CP, Herrera-Estrella A, Seidl-Seiboth V, Martinez DA, Druzhinina IS, Thon M, Zeilinger S, Casas-Flores S, Horwitz BA, Mukherjee PK, et al.. 2011. Comparative genome sequence analysis underscores mycoparasitism as the ancestral life style of Trichoderma. Genome Biol 12:R40. doi:10.1186/gb-2011-12-4-r40 21501500 PMC3218866

[4] Zeilinger S, Gruber S, Bansal R, Mukherjee PK. 2016. Secondary metabolism in Trichoderma – chemistry meets genomics. Fungal Biol Rev 30:74–90. doi:10.1016/j.fbr.2016.05.001

[5] Palmer JM, Keller NP. 2010. Secondary metabolism in fungi: does chromosomal location matter? Curr Opin Microbiol 13:431–436. doi:10.1016/j.mib.2010.04.008 20627806 PMC2922032

[6] Strauss J, Reyes-Dominguez Y. 2011. Regulation of secondary metabolism by chromatin structure and epigenetic codes. Fungal Genet Biol 48:62–69. doi:10.1016/j.fgb.2010.07.009 20659575 PMC3935439

[7] Bannister AJ, Kouzarides T. 2011. Regulation of chromatin by histone modifications. Cell Res 21:381–395. doi:10.1038/cr.2011.22 21321607 PMC3193420

[8] Cichewicz RH. 2010. Epigenome manipulation as a pathway to new natural product scaffolds and their congeners. Nat Prod Rep 27:11–22. doi:10.1039/b920860g 20024091 PMC2958777

[9] Brosch G, Loidl P, Graessle S. 2008. Histone modifications and chromatin dynamics: a focus on filamentous fungi. FEMS Microbiol Rev 32:409–439. doi:10.1111/j.1574-6976.2007.00100.x 18221488 PMC2442719

[10] Lai Y, Wang L, Zheng W, Wang S. 2022. Regulatory roles of histone modifications in filamentous fungal pathogens. J Fungi (Basel) 8:565. doi:10.3390/jof8060565 35736048 PMC9224773

[11] Bauer I, Graessle S. 2021. Fungal lysine deacetylases in virulence, resistance, and production of small bioactive compounds. Genes (Basel) 12:1470. doi:10.3390/genes12101470 34680865 PMC8535771

[12] Graessle S, Loidl P, Brosch G. 2001. Histone acetylation: plants and fungi as model systems for the investigation of histone deacetylases. Cell Mol Life Sci 58:704–720. doi:10.1007/pl00000894 11437232 PMC11337366

[13] Johnson CA, Turner BM. 1999. Histone deacetylases: complex transducers of nuclear signals. Semin Cell Dev Biol 10:179–188. doi:10.1006/scdb.1999.0299 10441071

[14] Pfannenstiel BT, Keller NP. 2019. On top of biosynthetic gene clusters: how epigenetic machinery influences secondary metabolism in fungi. Biotechnol Adv 37:107345. doi:10.1016/j.biotechadv.2019.02.001 30738111 PMC6685777

[15] de Ruijter AJM, van Gennip AH, Caron HN, Kemp S, van Kuilenburg ABP. 2003. Histone deacetylases (HDACs): characterization of the classical HDAC family. Biochem J 370:737–749. doi:10.1042/BJ20021321 12429021 PMC1223209

[16] Osorio-Concepción M, Cristóbal-Mondragón GR, Gutiérrez-Medina B, Casas-Flores S. 2017. Histone deacetylase HDA-2 regulates Trichoderma atroviride growth, conidiation, blue light perception, and oxidative stress responses. Appl Environ Microbiol 83:e02922-16. doi:10.1128/AEM.02922-16 27864177 PMC5244289

[17] Tribus M, Galehr J, Trojer P, Brosch G, Loidl P, Marx F, Haas H, Graessle S. 2005. HdaA, a major class 2 histone deacetylase of Aspergillus nidulans, affects growth under conditions of oxidative stress. Eukaryot Cell 4:1736–1745. doi:10.1128/EC.4.10.1736-1745.2005 16215180 PMC1265891

[18] Shwab EK, Bok JW, Tribus M, Galehr J, Graessle S, Keller NP. 2007. Histone deacetylase activity regulates chemical diversity in Aspergillus. Eukaryot Cell 6:1656–1664. doi:10.1128/EC.00186-07 17616629 PMC2043372

[19] Lee I, Oh J-H, Shwab EK, Dagenais TRT, Andes D, Keller NP. 2009. HdaA, a class 2 histone deacetylase of Aspergillus fumigatus, affects germination and secondary metabolite production. Fungal Genet Biol 46:782–790. doi:10.1016/j.fgb.2009.06.007 19563902 PMC2755195

[20] Maeda K, Izawa M, Nakajima Y, Jin Q, Hirose T, Nakamura T, Koshino H, Kanamaru K, Ohsato S, Kamakura T, Kobayashi T, Yoshida M, Kimura M. 2017. Increased metabolite production by deletion of an HDA1-type histone deacetylase in the phytopathogenic fungi, Magnaporthe oryzae (Pyricularia oryzae) and Fusarium asiaticum. Lett Appl Microbiol 65:446–452. doi:10.1111/lam.12797 28862744

[21] Studt L, Schmidt FJ, Jahn L, Sieber CMK, Connolly LR, Niehaus E-M, Freitag M, Humpf H-U, Tudzynski B. 2013. Two histone deacetylases, FfHda1 and FfHda2, are important for Fusarium fujikuroi secondary metabolism and virulence. Appl Environ Microbiol 79:7719–7734. doi:10.1128/AEM.01557-13 24096420 PMC3837819

[22] Rundlett SE, Carmen AA, Kobayashi R, Bavykin S, Turner BM, Grunstein M. 1996. HDA1 and RPD3 are members of distinct yeast histone deacetylase complexes that regulate silencing and transcription. Proc Natl Acad Sci U S A 93:14503–14508. doi:10.1073/pnas.93.25.14503 8962081 PMC26162

[23] Stahl E, Glatz A. 1982. Zur Farbreaktion der Anisaldehyd-Schwefelsäure als Reagenz in der Dünnschicht-Chromatographie. J Chromatogr A 240:518–521. doi:10.1016/S0021-9673(00)99634-1

[24] Schalamun M, Beier S, Hinterdobler W, Wanko N, Schinnerl J, Brecker L, Engl DE, Schmoll M. 2023. MAPkinases regulate secondary metabolism, sexual development and light dependent cellulase regulation in Trichoderma reesei. Sci Rep 13:1912. doi:10.1038/s41598-023-28938-w 36732590 PMC9894936

[25] Rispail N, Soanes DM, Ant C, Czajkowski R, Grünler A, Huguet R, Perez-Nadales E, Poli A, Sartorel E, Valiante V, Yang M, Beffa R, Brakhage AA, Gow NAR, Kahmann R, Lebrun M-H, Lenasi H, Perez-Martin J, Talbot NJ, Wendland J, Di Pietro A. 2009. Comparative genomics of MAP kinase and calcium-calcineurin signalling components in plant and human pathogenic fungi. Fungal Genet Biol 46:287–298. doi:10.1016/j.fgb.2009.01.002 19570501

[26] Schmoll M, Dattenböck C, Carreras-Villaseñor N, Mendoza-Mendoza A, Tisch D, Alemán MI, Baker SE, Brown C, Cervantes-Badillo MG, Cetz-Chel J, et al.. 2016. The genomes of three uneven siblings: footprints of the lifestyles of three Trichoderma species. Microbiol Mol Biol Rev 80:205–327. doi:10.1128/MMBR.00040-15 26864432 PMC4771370

[27] Gruber S, Zeilinger S. 2014. The transcription factor Ste12 mediates the regulatory role of the Tmk1 MAP kinase in mycoparasitism and vegetative hyphal fusion in the filamentous fungus Trichoderma atroviride. PLoS One 9:e111636. doi:10.1371/journal.pone.0111636 25356841 PMC4214791

[28] Zhao S, Liao X-Z, Wang J-X, Ning Y-N, Li C-X, Liao L-S, Liu Q, Jiang Q, Gu L-S, Fu L-H, Yan Y-S, Xiong Y-R, He Q-P, Su L-H, Duan C-J, Luo X-M, Feng J-X. 2019. Transcription factor Atf1 regulates expression of cellulase and xylanase genes during solid-state fermentation of ascomycetes. Appl Environ Microbiol 85:e01226-19. doi:10.1128/AEM.01226-19 31604764 PMC6881793

[29] Liu L, Tang M-C, Tang Y. 2019. Fungal highly reducing polyketide synthases biosynthesize salicylaldehydes that are precursors to epoxycyclohexenol natural products. J Am Chem Soc 141:19538–19541. doi:10.1021/jacs.9b09669 31790246 PMC6924165

[30] Brown DW, Lee S-H, Kim L-H, Ryu J-G, Lee S, Seo Y, Kim YH, Busman M, Yun S-H, Proctor RH, Lee T. 2015. Identification of a 12-gene fusaric acid biosynthetic gene cluster in Fusarium species through comparative and functional genomics. Mol Plant Microbe Interact 28:319–332. doi:10.1094/MPMI-09-14-0264-R 25372119

[31] Estrada-Rivera M, Rebolledo-Prudencio OG, Pérez-Robles DA, Rocha-Medina MDC, González-López MDC, Casas-Flores S. 2019. Trichoderma histone deacetylase HDA-2 modulates multiple responses in Arabidopsis. Plant Physiol 179:1343–1361. doi:10.1104/pp.18.01092 30670606 PMC6446751

[32] Li Y, Wang C, Liu W, Wang G, Kang Z, Kistler HC, Xu J-R. 2011. The HDF1 histone deacetylase gene is important for conidiation, sexual reproduction, and pathogenesis in Fusarium graminearum. Mol Plant Microbe Interact 24:487–496. doi:10.1094/MPMI-10-10-0233 21138346

[33] Ma H, Li L, Gai Y, Zhang X, Chen Y, Zhuo X, Cao Y, Jiao C, Gmitter FG, Li H. 2021. Histone acetyltransferases and deacetylases are required for virulence, conidiation, DNA damage repair, and multiple stresses resistance of Alternaria alternata. Front Microbiol 12:783633. doi:10.3389/fmicb.2021.783633 34880849 PMC8645686

[34] Izawa M, Takekawa O, Arie T, Teraoka T, Yoshida M, Kimura M, Kamakura T. 2009. Inhibition of histone deacetylase causes reduction of appressorium formation in the rice blast fungus Magnaporthe oryzae. J Gen Appl Microbiol 55:489–498. doi:10.2323/jgam.55.489 20118613

[35] Li X, Cai Q, Mei H, Zhou X, Shen Y, Li D, Liu W. 2015. The Rpd3/Hda1 family of histone deacetylases regulates azole resistance in Candida albicans. J Antimicrob Chemother 70:1993–2003. doi:10.1093/jac/dkv070 25825380

[36] Lan H, Wu L, Sun R, Keller NP, Yang K, Ye L, He S, Zhang F, Wang S. 2019. The HosA histone deacetylase regulates aflatoxin biosynthesis through direct regulation of aflatoxin cluster genes. Mol Plant Microbe Interact 32:1210–1228. doi:10.1094/MPMI-01-19-0033-R 30986121

[37] Griffiths S, Saccomanno B, de Wit P, Collemare J. 2015. Regulation of secondary metabolite production in the fungal tomato pathogen Cladosporium fulvum. Fungal Genet Biol 84:52–61. doi:10.1016/j.fgb.2015.09.009 26415644

[38] Rajani P, Rajasekaran C, Vasanthakumari MM, Olsson SB, Ravikanth G, Uma Shaanker R. 2021. Inhibition of plant pathogenic fungi by endophytic Trichoderma spp. through mycoparasitism and volatile organic compounds. Microbiol Res 242:126595. doi:10.1016/j.micres.2020.126595 33017769

[39] Alfiky A, Weisskopf L. 2021. Deciphering Trichoderma-plant-pathogen interactions for better development of biocontrol applications. J Fungi (Basel) 7:61. doi:10.3390/jof7010061 33477406 PMC7830842

[40] Ayyandurai M, Akila R, Manonmani K, Harish S, Mini ML, Vellaikumar S. 2023. Deciphering the mechanism of Trichoderma spp. consortia possessing volatile organic compounds and antifungal metabolites in the suppression of Sclerotium rolfsii in groundnut. Physiol Mol Plant Pathol 125:102005. doi:10.1016/j.pmpp.2023.102005

[41] Li N, Alfiky A, Wang W, Islam M, Nourollahi K, Liu X, Kang S. 2018. Volatile compound-mediated recognition and inhibition between Trichoderma biocontrol agents and Fusarium oxysporum. Front Microbiol 9:2614. doi:10.3389/fmicb.2018.02614 30455673 PMC6231246

[42] Yalage Don SM, Schmidtke LM, Gambetta JM, Steel CC. 2020. Aureobasidium pullulans volatilome identified by a novel, quantitative approach employing SPME-GC-MS, suppressed Botrytis cinerea and Alternaria alternata in vitro. Sci Rep 10:4498. doi:10.1038/s41598-020-61471-8 32161291 PMC7066187

[43] Herrera JM, Pizzolitto RP, Zunino MP, Dambolena JS, Zygadlo JA. 2015. Effect of fungal volatile organic compounds on a fungus and an insect that damage stored maize. J Stored Prod Res 62:74–80. doi:10.1016/j.jspr.2015.04.006

[44] Kaur M, Kumari A, Singh R. 2022. The indigenous volatile inhibitor 2-methyl-2-butene impacts biofilm formation and interspecies interaction of the pathogenic mucorale Rhizopus arrhizus. Microb Ecol 83:506–512. doi:10.1007/s00248-021-01765-0 34023922

[45] Kong W-L, Rui L, Ni H, Wu X-Q. 2020. Antifungal effects of volatile organic compounds produced by Rahnella aquatilis JZ-GX1 against Colletotrichum gloeosporioides in Liriodendron chinense × tulipifera. Front Microbiol 11:1114. doi:10.3389/fmicb.2020.01114 32547526 PMC7271530

[46] Ramin AA, Braun PG, Prange RK, DeLong JM. 2005. In vitro effects of Muscodor albus and three volatile components on growth of selected postharvest microorganisms. HortSci 40:2109–2114. doi:10.21273/HORTSCI.40.7.2109

[47] Dieryckx C, Gaudin V, Dupuy J-W, Bonneu M, Girard V, Job D. 2015. Beyond plant defense: insights on the potential of salicylic and methylsalicylic acid to contain growth of the phytopathogen Botrytis cinerea. Front Plant Sci 6:859. doi:10.3389/fpls.2015.00859 26528317 PMC4607878

[48] Wang Y, Zhu X, Wang J, Shen C, Wang W. 2023. Identification of mycoparasitism-related genes against the phytopathogen Botrytis cinerea via transcriptome analysis of Trichoderma harzianum T4. J Fungi (Basel) 9:324. doi:10.3390/jof9030324 36983492 PMC10055783

[49] Brandão F, Esher SK, Ost KS, Pianalto K, Nichols CB, Fernandes L, Bocca AL, Poças-Fonseca MJ, Alspaugh JA. 2018. HDAC genes play distinct and redundant roles in Cryptococcus neoformans virulence. Sci Rep 8:5209. doi:10.1038/s41598-018-21965-y 29581526 PMC5979944

[50] Kim T, Lee SH, Oh YT, Jeon J. 2020. A histone deacetylase, MoHDA1 regulates asexual development and virulence in the rice blast fungus. Plant Pathol J 36:314–322. doi:10.5423/PPJ.OA.06.2020.0099 32788890 PMC7403517

[51] Jeon J, Kwon S, Lee Y-H. 2014. Histone acetylation in fungal pathogens of plants. Plant Pathol J 30:1–9. doi:10.5423/PPJ.RW.01.2014.0003 25288980 PMC4174833

[52] Poças-Fonseca MJ, Cabral CG, Manfrão-Netto JHC. 2020. Epigenetic manipulation of filamentous fungi for biotechnological applications: a systematic review. Biotechnol Lett 42:885–904. doi:10.1007/s10529-020-02871-8 32246346

[53] Smith KM, Phatale PA, Bredeweg EL, Connolly LR, Pomraning KR, Freitag M. 2006. Epigenetics of filamentous fungi. In Meyers RA (ed), Encyclopedia of molecular cell biology and molecular medicine. Wiley-VCH Verlag GmbH & Co. KGaA, Weinheim, Germany.

[54] Robyr D, Suka Y, Xenarios I, Kurdistani SK, Wang A, Suka N, Grunstein M. 2002. Microarray deacetylation maps determine genome-wide functions for yeast histone deacetylases. Cell 109:437–446. doi:10.1016/s0092-8674(02)00746-8 12086601

[55] Helsen J, Voordeckers K, Vanderwaeren L, Santermans T, Tsontaki M, Verstrepen KJ, Jelier R. 2020. Gene loss predictably drives evolutionary adaptation. Mol Biol Evol 37:2989–3002. doi:10.1093/molbev/msaa172 32658971 PMC7530610

[56] Weiner A, Chen HV, Liu CL, Rahat A, Klien A, Soares L, Gudipati M, Pfeffner J, Regev A, Buratowski S, Pleiss JA, Friedman N, Rando OJ. 2012. Systematic dissection of roles for chromatin regulators in a yeast stress response. PLoS Biol 10:e1001369. doi:10.1371/journal.pbio.1001369 22912562 PMC3416867

[57] Wang S-H, Lin P-Y, Chiu Y-C, Huang J-S, Kuo Y-T, Wu J-C, Chen C-C. 2015. Curcumin-mediated HDAC inhibition suppresses the DNA damage response and contributes to increased DNA damage sensitivity. PLoS One 10:e0134110. doi:10.1371/journal.pone.0134110 26218133 PMC4517890

[58] Guzman-Chavez F, Salo O, Samol M, Ries M, Kuipers J, Bovenberg RAL, Vreeken RJ, Driessen AJM. 2018. Deregulation of secondary metabolism in a histone deacetylase mutant of Penicillium chrysogenum. Microbiologyopen 7:e00598. doi:10.1002/mbo3.598 29575742 PMC6182556

[59] Robbins N, Leach MD, Cowen LE. 2012. Lysine deacetylases Hda1 and Rpd3 regulate Hsp90 function thereby governing fungal drug resistance. Cell Rep 2:878–888. doi:10.1016/j.celrep.2012.08.035 23041319 PMC3607219

[60] Niehaus E-M, Studt L, von Bargen KW, Kummer W, Humpf H-U, Reuter G, Tudzynski B. 2016. Sound of silence: the beauvericin cluster in Fusarium fujikuroi is controlled by cluster-specific and global regulators mediated by H3K27 modification. Environ Microbiol 18:4282–4302. doi:10.1111/1462-2920.13576 27750383

[61] Godio RP, Martín JF. 2009. Modified oxidosqualene cyclases in the formation of bioactive secondary metabolites: biosynthesis of the antitumor clavaric acid. Fungal Genet Biol 46:232–242. doi:10.1016/j.fgb.2008.12.002 19130892

[62] Sasaki H, Hosokawa T, Sawada M, Ando K. 1973. Isolation and structure of ascofuranone and ascofranol, antibiotics with hypolipidemic activity. J Antibiot 26:676–680. doi:10.7164/antibiotics.26.676 4792115

[63] Ellestad GA, Evans RH, Kunstmann MP. 1969. Some new terpenoid metabolites from an unidentified Fusarium species. Tetrahedron 25:1323–1334. doi:10.1016/s0040-4020(01)82703-4 5346204

[64] Hamel L-P, Nicole M-C, Duplessis S, Ellis BE. 2012. Mitogen-activated protein kinase signaling in plant-interacting fungi: distinct messages from conserved messengers. Plant Cell 24:1327–1351. doi:10.1105/tpc.112.096156 22517321 PMC3398478

[65] de Nadal E, Posas F. 2008. Regulation of gene expression in response to osmostress by the yeast stress-activated protein kinase Hog1, p 81–97. In Posas F, Nebreda AR (ed), Stress-activated protein kinases. Vol. 20. Springer, Berlin Heidelberg.

[66] De Nadal E, Zapater M, Alepuz PM, Sumoy L, Mas G, Posas F. 2004. The MAPK Hog1 recruits Rpd3 histone deacetylase to activate osmoresponsive genes. Nature 427:370–374. doi:10.1038/nature02258 14737171

[67] Kawauchi M, Iwashita K. 2014. Functional analysis of histone deacetylase and its role in stress response, drug resistance and solid-state cultivation in Aspergillus oryzae. J Biosci Bioeng 118:172–176. doi:10.1016/j.jbiosc.2014.02.004 24613105

[68] Yu R, Cao X, Sun L, Zhu J-Y, Wasko BM, Liu W, Crutcher E, Liu H, Jo MC, Qin L, Kaeberlein M, Han Z, Dang W. 2021. Inactivating histone deacetylase HDA promotes longevity by mobilizing trehalose metabolism. Nat Commun 12:1981. doi:10.1038/s41467-021-22257-2 33790287 PMC8012573

[69] Speckbacher V, Ruzsanyi V, Martinez-Medina A, Hinterdobler W, Doppler M, Schreiner U, Böhmdorfer S, Beccaccioli M, Schuhmacher R, Reverberi M, Schmoll M, Zeilinger S. 2020. The lipoxygenase Lox1 is involved in light- and injury-response, conidiation, and volatile organic compound biosynthesis in the mycoparasitic fungus Trichoderma atroviride. Front Microbiol 11:2004. doi:10.3389/fmicb.2020.02004 32973724 PMC7482316

[70] Tamura K, Stecher G, Kumar S, Battistuzzi FU. 2021. MEGA11: molecular evolutionary genetics analysis version 11. Mol Biol Evol 38:3022–3027. doi:10.1093/molbev/msab120 33892491 PMC8233496

[71] Hartl L, Kubicek CP, Seiboth B. 2007. Induction of the gal pathway and cellulase genes involves no transcriptional inducer function of the galactokinase in Hypocrea jecorina. J Biol Chem 282:18654–18659. doi:10.1074/jbc.M700955200 17452322

[72] Moreno-Ruiz D, Salzmann L, Fricker MD, Zeilinger S, Lichius A. 2021. Stress-activated protein kinase signalling regulates mycoparasitic hyphal-hyphal interactions in Trichoderma atroviride. J Fungi (Basel) 7:365. doi:10.3390/jof7050365 34066643 PMC8148604

[73] Catlett NL, Lee B-N, Yoder OC, Turgeon BG. 2003. Split-marker recombination for efficient targeted deletion of fungal genes. Fungal Genet Rep 50:9–11. doi:10.4148/1941-4765.1150

[74] Gruber F, Visser J, Kubicek CP, de Graaff LH. 1990. The development of a heterologous transformation system for the cellulolytic fungus Trichoderma reesei based on a pyrG-negative mutant strain. Curr Genet 18:71–76. doi:10.1007/BF00321118 2245476

[75] Brunner K, Omann M, Pucher ME, Delic M, Lehner SM, Domnanich P, Kratochwill K, Druzhinina I, Denk D, Zeilinger S. 2008. Trichoderma G protein-coupled receptors: functional characterisation of a cAMP receptor-like protein from Trichoderma atroviride. Curr Genet 54:283–299. doi:10.1007/s00294-008-0217-7 18836726 PMC2855678

[76] Steyaert J, Hicks E, Kandula J, Kandula D, Alizadeh H, Braithwaite M, Yardley J, Mendoza-Mendoza A, Stewart A. 2016. Methods for the evaluation of the bioactivity and biocontrol potential of species of *Trichoderma*, p 23–35. In Glare TR, Moran-Diez ME (ed), Microbial-based biopesticides. Methods and protocols. Humana Press, New York, NY. 10.1007/978-1-4939-6367-6_3 27565489

[77] Speckbacher V, Zeilinger S, Zimmermann S, Mayhew CA, Wiesenhofer H, Ruzsanyi V. 2021. Monitoring the volatile language of fungi using gas chromatography-ion mobility spectrometry. Anal Bioanal Chem 413:3055–3067. doi:10.1007/s00216-021-03242-6 33675374 PMC8043876

[78] Yan L. 2021. ggvenn: draw venn diagram by 'ggplot2'. Available from: https://CRAN.R-project.org/package=ggvenn

[79] Kolde R. 2019. pheatmap: pretty heatmaps. Available from: https://CRAN.R-project.org/package=pheatmap

[80] Conesa A, Götz S, García-Gómez JM, Terol J, Talón M, Robles M. 2005. Blast2GO: a universal tool for annotation, visualization and analysis in functional genomics research. Bioinformatics 21:3674–3676. doi:10.1093/bioinformatics/bti610 16081474

[81] Paysan-Lafosse T, Blum M, Chuguransky S, Grego T, Pinto BL, Salazar GA, Bileschi ML, Bork P, Bridge A, Colwell L, et al.. 2023. InterPro in 2022. Nucleic Acids Res 51:D418–D427. doi:10.1093/nar/gkac993 36350672 PMC9825450

[82] UniProt Consortium. 2023. UniProt: the universal protein knowledgebase in 2023. Nucleic Acids Res 51:D523–D531. doi:10.1093/nar/gkac1052 36408920 PMC9825514

[83] Supek F, Bošnjak M, Škunca N, Šmuc T. 2011. REVIGO summarizes and visualizes long lists of gene ontology terms. PLoS One 6:e21800. doi:10.1371/journal.pone.0021800 21789182 PMC3138752

[84] Kassambara A. 2022. ggpubr: 'ggplot2' based publication ready plots. Available from: https://cran.r-project.org/web/packages/ggpubr/index.html

[85] Kanehisa M, Furumichi M, Sato Y, Kawashima M, Ishiguro-Watanabe M. 2023. KEGG for taxonomy-based analysis of pathways and genomes. Nucleic Acids Res 51:D587–D592. doi:10.1093/nar/gkac963 36300620 PMC9825424

[86] Bu D, Luo H, Huo P, Wang Z, Zhang S, He Z, Wu Y, Zhao L, Liu J, Guo J, Fang S, Cao W, Yi L, Zhao Y, Kong L. 2021. KOBAS-i: intelligent prioritization and exploratory visualization of biological functions for gene enrichment analysis. Nucleic Acids Res 49:W317–W325. doi:10.1093/nar/gkab447 34086934 PMC8265193

[87] Wickham H. 2016. ggplot2: elegant graphics for data analysis. Use R! 2nd ed. Springer, Cham.

[88] Blin K, Shaw S, Kloosterman AM, Charlop-Powers Z, van Wezel GP, Medema MH, Weber T. 2021. antiSMASH 6.0: improving cluster detection and comparison capabilities. Nucleic Acids Res 49:W29–W35. doi:10.1093/nar/gkab335 33978755 PMC8262755

[89] Tribus M, Bauer I, Galehr J, Rieser G, Trojer P, Brosch G, Loidl P, Haas H, Graessle S. 2010. A novel motif in fungal class 1 histone deacetylases is essential for growth and development of Aspergillus. Mol Biol Cell 21:345–353. doi:10.1091/mbc.e09-08-0750 19940017 PMC2808227

[90] Trojer P, Brandtner EM, Brosch G, Loidl P, Galehr J, Linzmaier R, Haas H, Mair K, Tribus M, Graessle S. 2003. Histone deacetylases in fungi: novel members, new facts. Nucleic Acids Res 31:3971–3981. doi:10.1093/nar/gkg473 12853613 PMC167634

