# Supplementary material for: The histone deacetylase Hda1 affects oxidative and osmotic stress response as well as mycoparasitic activity and secondary metabolite biosynthesis in Trichoderma atroviride
Source: Microbiol Spectr. 2024 Feb 9;12(3):e03097-23. doi: 10.1128/spectrum.03097-23 (PMC10913545; doi:10.1128/spectrum.03097-23)
Supplement: Supplemental figures and tables — Fig. S1 to S5; Tables S1 to S5. [file spectrum.03097-23-s0001.pdf]

SUPPLEMENTARY INFORMATION

Supplementary Table 1 – Identification of the *T. atroviride* Hda1 orthologue via BLASTp analysis. The *T. atroviride* orthologue of functionally characterized Hda1/HdaA proteins of *F. fujikuroi, M. oryzae, A. alternata, A, nidulans, C. fulvum, S. cerevisiae* and *A. fumigatus* was identified via BLASTp analysis (https://mycocosm.jgi.doe.gov/Triatrov1/Triatrov1.home.html). The top hit of BLASTp analysis is shown and sorted in descending order of the alignment score.

**Species**<br>Protein ID	**Score**	**Evalue**	**% Identity**	**% Subj Cov**	**Upmost Hit**
Fusarium fujikuroi<br>XP_023435500.1	3,022	0.00E000	97.6	92.6	Triatrov1_386002
Magnaporthe oryzae<br>XP_003717862.1	2,546	0.00E000	67.5	92.5	Triatrov1_386002
Alternaria alternata<br>RYN77957.1	1,670	0.00E000	51.0	79.9	Triatrov1_386002
Aspergillus nidulans<br>**XP_050467238.1**	1,571	1.52E-169	55.5	70.0	Triatrov1_386002
Cladosporium fulvum<br>Clafu1_192224	1,532	6.03E-178	53.7	70.3	Triatrov1_386002
Saccharomyces cerevisiae<br>NP_014377.1	1,311	1.58E-128	48.1	65.9	Triatrov1_386002
Aspergillus fumigatus<br>XP_748144.1	1,270	1.22E-127	56.0	57.9	Triatrov1_386002

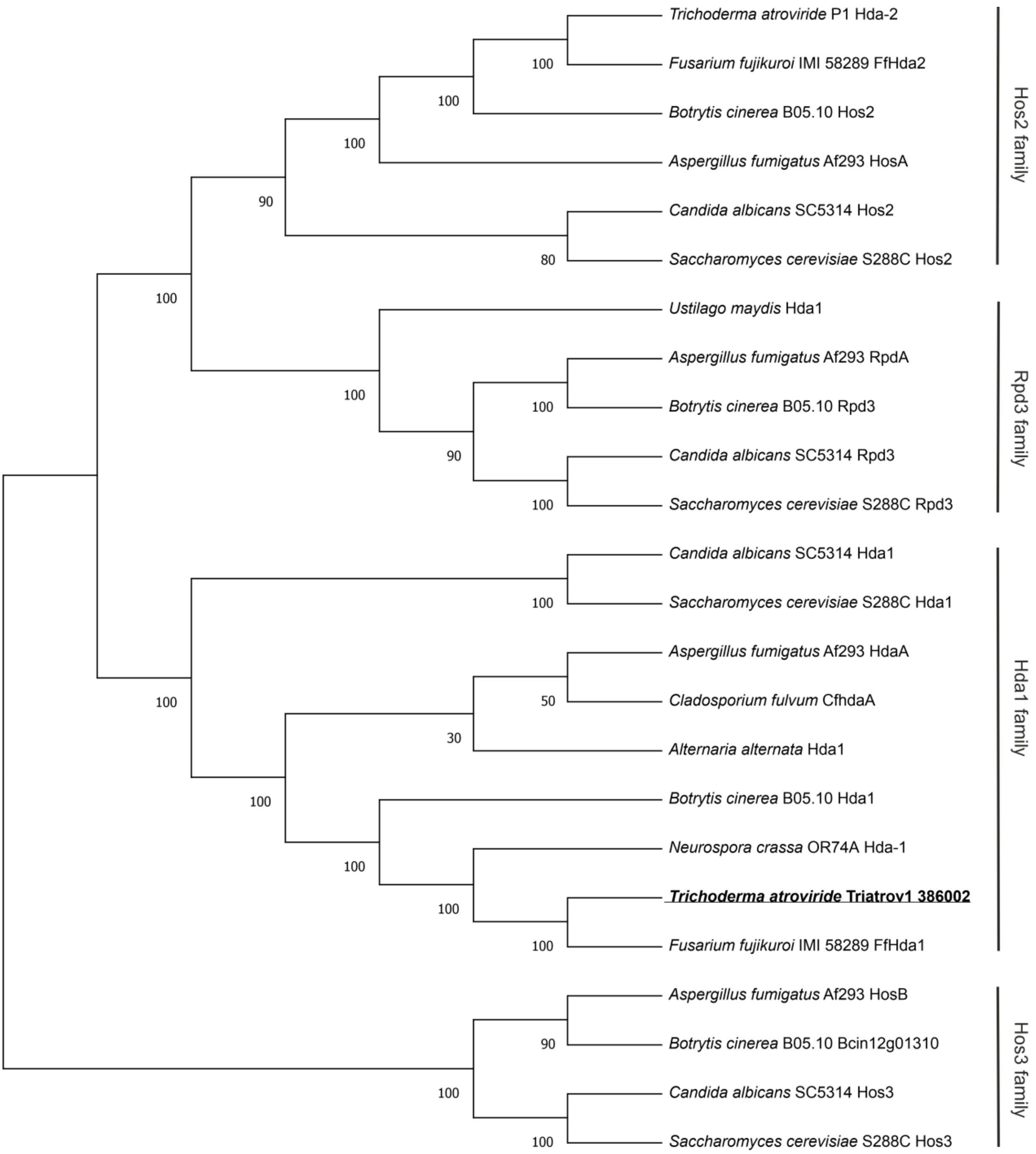

Supplementary Figure 1 – Analysis of the phylogenetic relationship of *T. atroviride* Hda1. The unrooted phylogenetic tree was constructed by comparing amino acid sequences of HDACs of different fungal species using the maximum likelihood algorithm of MEGA11. Numbers indicate the bootstrap probability values of observing the branch topology shown. Predicted Hda1 of *T. atroviride* is highlighted in bold. Information on proteins used in phylogenetic tree construction is provided in Materials and Methods section.

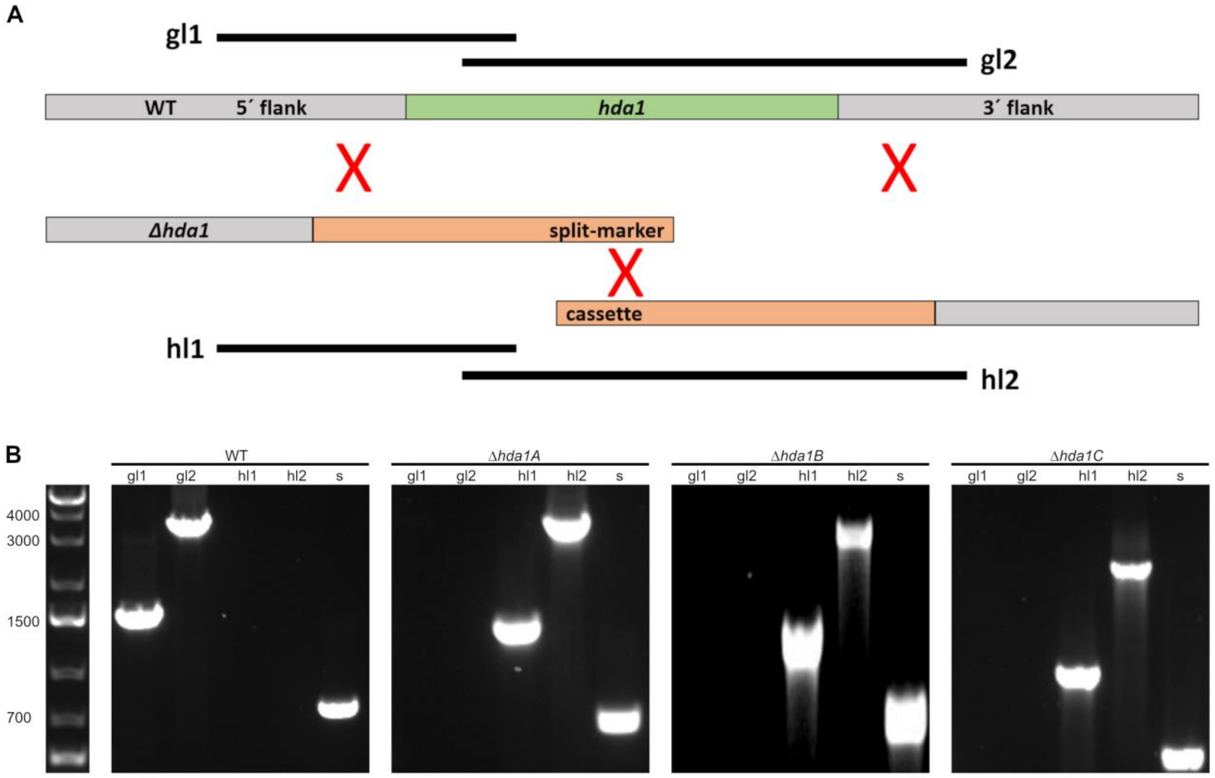

Supplementary Figure 2 – PCR genotyping analysis of *T. atroviride* wild type and the three independent ∆*hda1* mutants. (A) PCR genotyping strategy applied for screening the mitotically stable *hda1 d*eletion mutants after three rounds of single spore isolation: The successful deletion and hence absence of the *hda1* target locus in the deletion mutants was verified with the two *hda1*-locus specific primer pairings gl1 (1540 bp) and gl2 (3443 bp). The locus-specific integration and hence presence of the split-marker deletion cassette was verified with the two cassette-specific primer pairings hl1 (1461 bp) and hl2 (3456 bp). **(B)** Genotyping gels of the wildtype control (WT) and the three independent *hda1* deletion mutants (∆*hda1A,* ∆*hda1B,* ∆*hda1C*). S*ar1* (s, 712 bp) was targeted as internal control in all samples.

Supplementary Table 2 – Details of statistical analysis given in the figures

Factor	Degree of Freedom	Significance	Factor	Group	Significance
**Figure 2A - Growth Rate from Mycelia and Spores**					
	2-Way ANOVA			Bonferroni posttest	
Interaction	3	**	mycelia	WT - Δhda1A	ns
Inoculation Type	1	****		WT - Δhda1B	ns
Strain	3	**		WT - Δhda1C	ns
Residual	24			Δhda1A - Δhda1B	ns
				Δhda1A - Δhda1C	ns
				Δhda1B - Δhda1C	ns
			spores	WT - Δhda1A	***
				WT - Δhda1B	***
				WT - Δhda1C	***
				Δhda1A - Δhda1B	ns
				Δhda1A - Δhda1C	ns
				Δhda1B - Δhda1C	ns
**Figure 2B - Germination rate**					
	1-Way-ANOVA			Bonferroni`s multiple comparison	
**8 h:** strains	3	***	8 h	WT - Δhda1A	***
samples	8			WT - Δhda1B	***
total	11			WT - Δhda1C	***
**9 h:** strains	3	ns	9 h	WT - Δhda1A	ns
samples	8			WT - Δhda1B	ns
total	11			WT - Δhda1C	ns
**10 h:** strains	3	ns	10 h	WT - Δhda1A	ns
samples	8			WT - Δhda1B	ns
total	11			WT - Δhda1C	ns
**11 h:** strains	3	ns	11 h	WT - Δhda1A	ns
samples	8			WT - Δhda1B	ns
total	11			WT - Δhda1C	ns
**12 h:** strains	3	ns	12 h	WT - Δhda1A	ns
samples	8			WT - Δhda1B	ns
total	11			WT - Δhda1C	ns
**Figure 4 - Inhibition Index**					
	1-Way ANOVA			Tukey´s multiple comparison	
strains	3	**	strains	WT - Δhda1A	**
samples	11			WT - Δhda1B	*
total	14			WT - Δhda1C	**
				Δhda1A - Δhda1B	ns
				Δhda1A - Δhda1C	ns
				Δhda1B - Δhda1C	ns
**Figure 5 - VOC**					

2-methyl-butanol (Fig. 5A)	2-Way ANOVA			Šídák's multiple comparisons	
Interaction	8	****	WT - Δhda1A	21	ns
Incubation Time	8	****		24.5	ns
Strain	1	****		43.5	ns
Residual	54			48.5	ns
				68.5	ns
				72.5	**
				91.5	****
				96.5	****
				115.5	ns
**3-methyl butanol (Fig. 5B)**	2-Way ANOVA			Šídák's multiple comparisons	
Interaction	8	****	WT - Δhda1A	21	ns
Incubation Time	8	****		24.5	ns
Strain	1	****		43.5	ns
Residual	54			48.5	ns
				68.5	**
				72.5	****
				91.5	*
				96.5	**
				115.5	ns
**ethanol (Fig. 5C)**	2-Way ANOVA			Šídák's multiple comparisons	
Interaction	8	****	WT - Δhda1A	21	ns
Incubation Time	8	****		24.5	ns
Strain	1	****		43.5	ns
Residual	54			48.5	ns
				68.5	****
				72.5	****
				91.5	ns
				96.5	ns
				115.5	ns
**3-octanone (Fig. 5D)**	2-Way ANOVA			Šídák's multiple comparisons	
Interaction	8	****	WT - Δhda1A	21	ns
Incubation Time	8	****		24.5	ns
Strain	1	****		43.5	ns
Residual	54			48.5	ns
				68.5	ns
				72.5	ns
				91.5	ns
				96.5	ns
				115.5	****
**Figure 6 - Stress Assay**					
	2-Way ANOVA			Bonferroni posttest	

Interaction	12	***	PDA	WT - Δhda1A	***
Strain	3	***		WT - Δhda1B	**
Stress Factor	4	***		WT - Δhda1C	***
Residual	55		Sorbitol	WT - Δhda1A	***
				WT - Δhda1B	***
				WT - Δhda1C	***
			NaCl	WT - Δhda1A	***
				WT - Δhda1B	***
				WT - Δhda1C	**
			Menadione	WT - Δhda1A	ns
				WT - Δhda1B	***
				WT - Δhda1C	ns
			H2O2	WT - Δhda1A	***
				WT - Δhda1B	***
				WT - Δhda1C	***
**Supplementary Figure 3 - Dry Weight**					
	2-Way ANOVA			Šídák's multiple comparisons	
Interaction	1	ns	WT - Δhda1A	PDB only	****
Strain	1	****		PDB + Sorbitol	****
Medium	1	ns			
Residual	16				

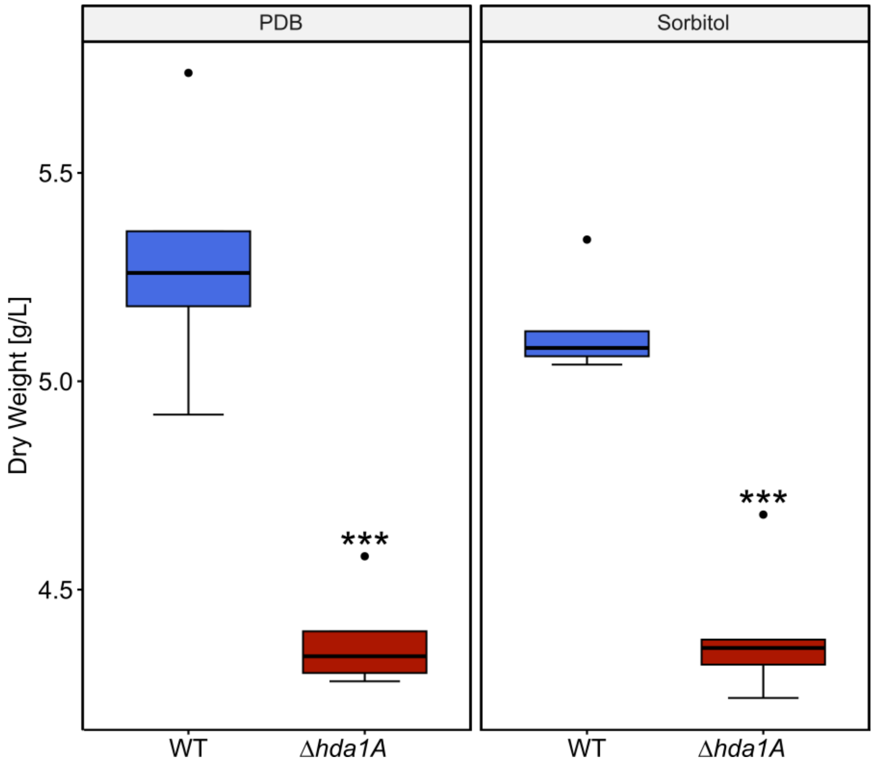

Supplementary Figure 3 – Biomass production of the Δ*hda1A* mutant and the wild type in liquid culture in the presence and absence of sorbitol. Mycelial dry weight [g DW/L] of the wild type (WT) and the Δ*hda1A* mutant upon cultivation in potato dextrose broth in the absence (PDB) and presence of sorbitol (Sorbitol). Strains were cultivated at 25°C and 250 rpm for a total time span of 54 h. Sorbitol (50 mM) was added as a single pulse after 30 h of incubation. Asterisks indicate statistically significant differences between the Δ*hda1* mutant and the WT within each treatment group (n = 5, *: $p < 0.05$, **: $p < 0.01$, ***: $p < 0.001$, ****: $p < 0.0001$; details of the statistical evaluation are given in Supplementary Table 2).

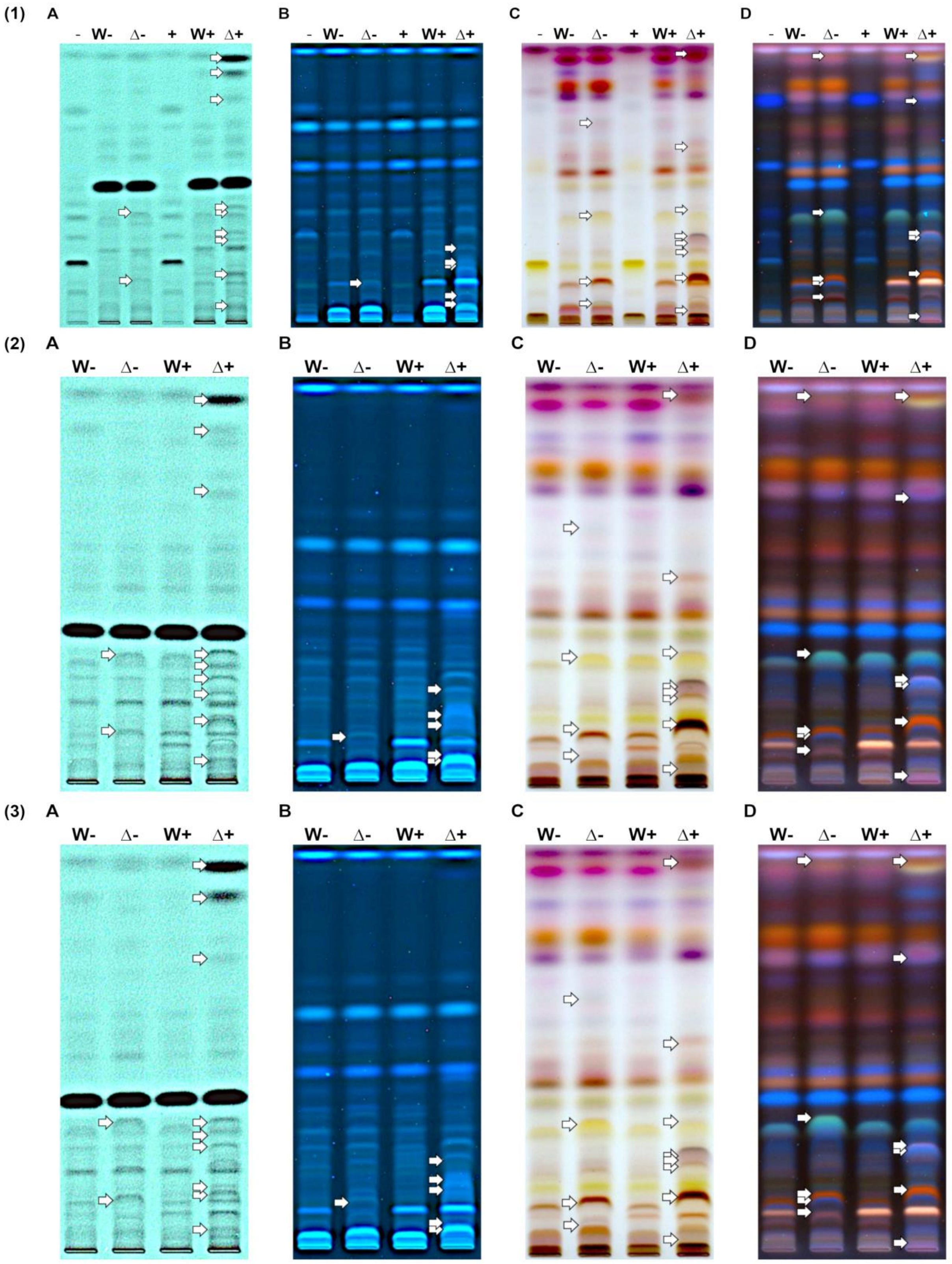

Supplementary Figure 4 – HPTLC fingerprint of metabolites secreted by the Δ*hda1A* mutant and the wild type upon growth in PDB in the presence and absence of sorbitol. HPTLC fingerprints of substances extracted from supernatants of replicates 2 to 4 (row (1) to (3)) of liquid cultures. The wildtype (W) and Δ*hda1A* (Δ)

were incubated in potato dextrose broth in the absence (-) and presence of sorbitol (+). In replicate 2 (1) pure potato dextrose broth in the absence (-) and presence of sorbitol (+) were added as a control. Strains were cultivated at 25°C and 250 rpm for a total time span of 54 h. Sorbitol (50 mM) was added as a single pulse after 30 h of incubation. Metabolites were extracted from culture supernatants and subjected to HPTLC analysis. Photos were taken at 254 nm (remission; UV absorbing substances; A), white light (transmission; visible substances; C) and 366 nm (remission; fluorescent substances; B, D) before (A, B) and after (C, D) derivatization with p-anisaldehyde sulfuric acid reagent. White arrows highlight differences (bands with higher intensity and additional bands) in the metabolite fingerprints of the Δ*hda1A* mutant compared to the WT.

Supplementary File A – List of DEGs of the four comparisons S1, S2, S3, S4. DEGs of the Δ*hda1A* mutant *versus* the wild type upon growth in PDB in the absence (S1) or presence (S2) of sorbitol. DEGs of the wild type grown in the presence of sorbitol *versus* grown in untreated PDB (S3) and *Δhda1A* mutant grown in the presence of sorbitol *versus* grown in untreated PDB (S4). Significantly up-regulated genes ($\log_2 FC \geq 1$) are highlighted in green; significantly down-regulated genes ($\log_2 FC \leq -1$) are highlighted in red.

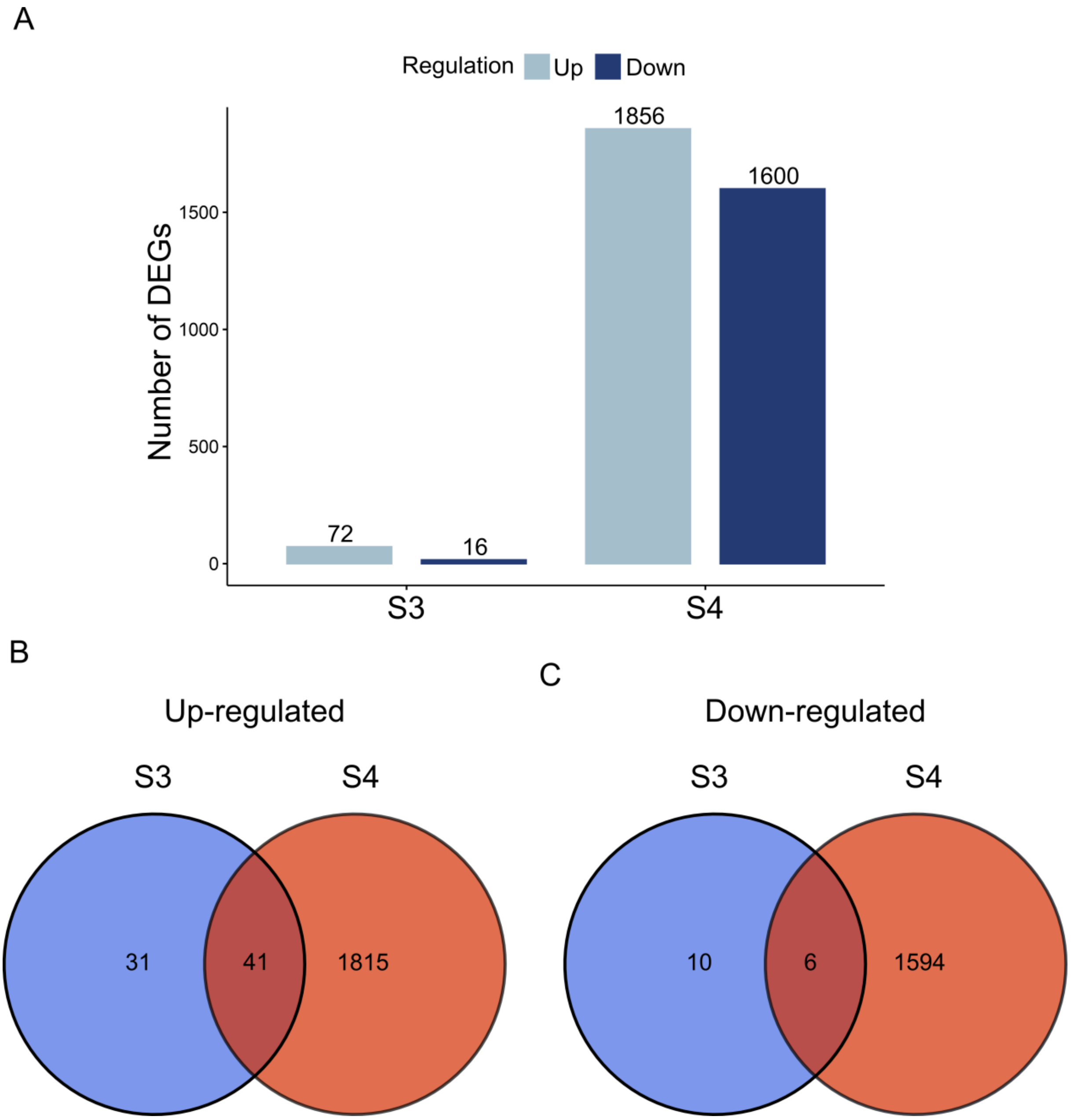

Supplementary Figure 5 – Comparison of differentially expressed genes (DEGs) in the *T. atroviride* WT and the Δ*hda1A* mutant upon growth in PDB in the presence *versus* absence of sorbitol. DEGs of the WT (S3) and the *Δhda1*A mutant (S4) grown in the presence of sorbitol *versus* grown in untreated PDB. DEGs with adjusted p-values ≤ 0.05 and absolute fold change values of $\log_2$FC ≥ 1 were considered either up- or down-regulated. **(A)** Distribution of up- and down-regulated DEGs between comparisons. The number of DEGs are indicated above the bars. Venn diagrams of up- **(B)** and down- **(C)** regulated DEGs of S3 and S4. The non-overlapping

regions represent the number of DEGs unique to each comparison. Overlapping regions represent the number of DEGs shared by the comparison groups.

Supplementary File B – GO and KEGG analyses of DEGs between the *Δhda1A* mutant *versus* the WT upon cultivation in PDB (S1). "GO Analysis" represents significantly enriched GO terms (FDR ≤ 0.05) in the *Δhda1A* mutant compared to the WT grown in untreated PDB, and "Gene Count" equals the number of genes assigned to a GO term. Up-regulated GO terms (DEGs with log2FC > 0) and down-regulated GO terms (DEGs with log2FC < 0) are highlighted in green and red, respectively. "KEGG" represents functional classification of up-regulated DEGs (log2FC > 0, highlighted in green) and down-regulated DEGs (log2FC < 0, highlighted in red) in the *Δhda1A* mutant compared to the WT grown in untreated PDB. Enrichment Ratio equals the number of observed (Input) divided by the number of expected (Background) genes from each KEGG category in the gene list.

Supplementary File C – GO and KEGG analysis of DEGs between the *Δhda1A* mutant *versus* the WT upon sorbitol treatment (S2). "GO Analysis" represents significantly enriched GO terms (FDR ≤ 0.05) in the *Δhda1A* mutant compared to the WT under osmotic stress conditions, and "Gene Count" equals the number of genes assigned to a GO term. Up-regulated GO terms (DEGs with log2FC > 0) and down-regulated GO terms (DEGs with log2FC < 0) are highlighted in green and red, respectively. "KEGG" represents functional classification of up-regulated DEGs (log2FC > 0, highlighted in green) and down-regulated DEGs (log2FC < 0, highlighted in red) in the *Δhda1A* mutant compared to the WT under osmotic stress conditions. "Enrichment Ratio" equals the number of observed (Input) divided by the number of expected (Background) genes from each KEGG category in the gene list. "BGCs"

represents up-regulated DEGs (log2FC > 0, highlighted in green) and down-regulated DEGs (log2FC < 0, highlighted in red) located in biosynthetic gene clusters and “TFs” represents the list of up-regulated and down-regulated transcription factors in the *Δhda1A* mutant compared to the WT under osmotic stress conditions

Supplementary Table 3 – Up-regulated DEGs in the comparison of the *T. atroviride Δhda1A* mutant *versus* the WT upon cultivation in PDB (S1) that are located in biosynthetic gene clusters (BGCs). DEGs with adjusted p-values ≤ 0.05 and fold change values of $\log_2$FC ≥ 1 were considered. Genes located in BGCs according to the genome database of *T. atroviride* IMI 206040.

Gene ID	Type	Cluster (JGI)	Cluster Type
**TRIATDRAFT_158537**	Oxidoreductase	Triat2.32	NRPS
**TRIATDRAFT_54918**	Phosphoprotein	Triat2.33	NRPS-Like
**TRIATDRAFT_291155**	MFS Transporter	Triat2.3	PKS
**TRIATDRAFT_256104**	Oxidoreductase	Triat2.3	PKS
**TRIATDRAFT_318140**	NAD(P)-binding	Triat2.11	PKS
**TRIATDRAFT_33143**	Lactamase B Domain	Triat2.18	TC

Supplementary Table 4 – Transcription factor (TF) genes differentially expressed in the *T. atroviride Δhda1A* mutant compared to the WT upon cultivation in PDB (S1). DEGs with adjusted p-values ≤ 0.05 and absolute fold change values of $\log_2$FC ≥ 1 were considered either up- or down-regulated. TF-encoding genes detected among the significantly regulated DEGs of the *Δhda1A* mutant compared to the WT cultivated in PDB.

Gene ID	Transcription factor family	Regulation
**TRIATDRAFT_298089**	C_2H_2 zinc finger	Up
**TRIATDRAFT_224048**	$Zn(II)_2Cys_6$ type	Up
**TRIATDRAFT_291139**	$Zn(II)_2Cys_6$ type	Up
**TRIATDRAFT_131874**	C_2H_2 zinc finger	Up
**TRIATDRAFT_178083**	$Zn(II)_2Cys_6$ type	Up
**TRIATDRAFT_217124**	$Zn(II)_2Cys_6$ type	Up
**TRIATDRAFT_173455**	$Zn(II)_2Cys_6$ type	Up
**TRIATDRAFT_288678**	C_2H_2 zinc finger	Up
**TRIATDRAFT_4050**	$Zn(II)_2Cys_6$ type	Up
**TRIATDRAFT_54658**	bZIP	Down

Supplementary Table 5 – Primers used in this study

primer name	5´-3´ sequence
hda1_39952-5KO-F1	TAGGGGTTCCGCGCACATTTCCCCGACTATGCTATGCTGATGGTGG
hda1_39952-5KO-R1	TAAGGTAGCTCTCTGCATTTATTGTTATGCTTTTGC
hda1_39952-hph-F1	AACAATAAATGCAGAGAGCTACCTTACATCAATATGGC
hda1_39952-hph-R1	TGAGGACCAGATCGGTACTATGGCTTAGATGGAATACC
hda1_39952-3KO-F1	AAGCCATAGTACCGATCTGGTCCTCAGGCTC
hda1_39952-3KO-R1	CCTTTGAGTGAGCTGATACCGCTCGTGCTCTTTGCACGCAGAG
hda1_39952-HY-F1	ACTATGCTATGCTGATGGTGG
hda1_39952-YG-R1	TGCTCTTTGCACGCAGAG
hph-pLS3-CRIBc-F1	GAGAGCTACCTTACATCAATATGGC
hph-pLS3-CRIBc-R1	GGTATTCCATCTAAGCCATAGTACC
Catlett HY-R	GGATGCCTCCGCTCGAAGTA
Catlett YG-F	CGTTGCAAGACCTGCCTGAA
pRITA-bb-F1	CGAGCGGTATCAGCTCACTC
pRITA-bb-R1	CGGGGAAATGTGCGC
hda1_39952_C_F1	GCCGGGAAGACTTTTCGGTA
hda1_39952_C_R1	GCTTCCGTGCCAGATCGATA
hda1_39952_RT_F1	CGAAATCATCCTGCTTGGCG
hda1_39952_RT_R1	GCTCGTTATCCGTGTCCGAT
Pgapdh_hph_RT_F1	AAGGTGGTCATGCAGTTGCT
Pgapdh_hph_RT_R1	CGGATGAGCGACATTCATGC
301975_hkC_F_Brunner2008	CTCGACAATGCCGGAAAGACCA
301975_hkC_R_Brunner2008	TTGCCAAGGATGACAAAGGGG
